# Prognostic integration of tumor microenvironment and parthanatos-related genes in gastric cancer: a machine learning-driven risk model and immune landscape profiling

**DOI:** 10.3389/fimmu.2026.1636331

**Published:** 2026-02-20

**Authors:** Lei Liu, Min Wu, Yi Liu

**Affiliations:** 1 Central Laboratory, Shaanxi Provincial People’s Hospital, Xi’an, Shaanxi, China; 2 Department of Oncology, Shaanxi Provincial People’s Hospital, Xi’an, Shaanxi, China

**Keywords:** gastric cancer, tumor microenvironment, parthanatos, prognostic model, immune infiltration

## Abstract

**Background:**

The tumor microenvironment (TME) plays a pivotal role in the progression of gastric cancer (GC) and its response to treatment, particularly by modulating parthanatos (PA). However, the prognostic significance of TME and PA, as well as their potential roles in immunotherapy for GC, remain incompletely understood.

**Methods:**

Using publicly available data, an initial gene screening was combined with differential expression analysis and univariate Cox regression to identify prognostic markers associated with TME and PA. A comprehensive machine learning framework, testing 101 algorithm combinations across 10 methodologies, was then applied. Model selection prioritized C-index performance, with the final model enabling effective patient risk stratification, as validated by Receiver Operating Characteristic (ROC) curves. Multivariate analysis subsequently identified independent prognostic factors, which were used to construct a clinical nomogram. Immune characteristics across different risk groups were compared, immunofluorescence staining of gastric cancer and paired paracancerous tissues assessed immune cell infiltration and prognostic gene-monocyte correlation. Biomarker expression patterns were confirmed *via* Reverse Transcription Quantitative Polymerase Chain Reaction (RT-qPCR) validation. Immunohistochemical (IHC) detected *CD36*/*KIT* protein expression. CCK-8, Transwell and flow cytometry evaluated proliferation, migration and apoptosis in *CD36*/*KIT* inhibitor-treated GC MKN45 cells. Enzyme-linked immunosorbent assay (ELISA) measured TNF-α, IFN-γ, IL-10 secretion; immunofluorescence determined PARP-1/AIFM1 subcellular localization post-inhibitor treatment.

**Results:**

The multi-algorithm analysis identified the RSF-plsRcox hybrid model as the most accurate, consistently achieving C-index values greater than 0.6 across all datasets. This model identified seven clinically significant genes (*EGF*, *PCOLCE2*, *CD36*, *ADAMTS8*, *CIDEC*, *KIT*, and *AKAP12*) with notable prognostic value in GC progression. The developed risk model demonstrated good predictive performance, with areas under the curve (AUCs) of 0.65, 0.68, and 0.60 at 1, 3, and 5 years, respectively. A nomogram based on independent prognostic factors—risk scores, age, N stage, and M stage—showed strong predictive accuracy, with AUCs of 0.72, 0.75, and 0.71 at 1, 3, and 5 years, respectively. Furthermore, distinct immune landscapes were observed between high-risk and low-risk groups, characterized by differences in immune infiltration, immune checkpoint expression, and TIDE scores. Gastric cancer tissues had reduced CD3^+^T, CD3^+^CD4^+^T cells and M1 macrophage infiltration, increased M2 macrophages and CD14^+^monocytes; AKAP12 was positively correlated with monocytes. The higher-risk group exhibited suppressed immune responses and enhanced immune evasion capabilities. RT-qPCR validation revealed significant differential expression of *PCOLCE2*, *CD36*, *ADAMTS8*, and *KIT* in the control group, while *AKAP12* was more highly expressed in the GC group. These five prognostic genes showed significant expression differences between the two groups (*P* < 0.05).*CD36* and *KIT* protein expression was elevated in gastric cancer tissues. *CD36/KIT* inhibition downregulated their expression in MKN45 cells, inhibiting proliferation, migration and promoting apoptosis. Inhibition increased TNF-α, IFN-γ secretion, decreased IL-10, enhanced nuclear PARP-1 fluorescence and AIFM1 nuclear translocation.

**Conclusions:**

This study innovatively integrated TME-RGs and PA-RGs to construct a machine learning GC prognostic model (7 key genes: *EGF*, *PCOLCE2*, *CD36*, *ADAMTS8*, *CIDEC*, *KIT*, and *AKAP12*). High-risk patients had immunosuppressive TME and poor immunotherapy response, with Imatinib/PLX4720 showing potential efficacy. *CD36/KIT* overexpression promoted GC malignancy; their inhibition remodeled TME cytokines and, for the first time, activated the PA pathway to induce GC cell death.

## 1 Introduction

Gastric carcinoma (GC) is a molecularly heterogeneous group of malignancies originating from the gastric epithelium, characterized by significant pathological diversity and a multifactorial etiology. As a primary epithelial neoplasm of the stomach, its development results from complex interactions between genetic predisposition and environmental factors. GC is a complex and heterogeneous disease with multiple risk factors (1). According to the 2022 global cancer statistics, GC accounted for over 968,000 new cases and nearly 660,000 deaths, making it the fifth leading cause of cancer-related morbidity and mortality worldwide (2, 3). The development of GC is influenced by a combination of environmental and genetic risk factors, including Helicobacter pylori infection, alcohol consumption, smoking, high salt and pickled food intake, low fruit consumption, and genetic predisposition (4, 5). GC often lacks distinctive clinical features in its early stages, resulting in delayed diagnosis for most patients until the disease has advanced, often with metastasis (6). Despite advancements in the diagnosis and treatment of GC, including surgical resection, chemotherapy, radiotherapy, and targeted therapies, the overall efficacy remains suboptimal, and survival rates are low, particularly for patients with advanced GC, leading to a poor prognosis (7). Additionally, the high cost of treatment places a substantial economic burden on patients and their families, imposing significant pressure on public healthcare resources (7). The pathogenesis of GC is driven by various complex molecular and genetic alterations, including but not limited to dysregulation in cell proliferation, apoptosis, angiogenesis, and immune evasion (4, 5). Thus, investigating the molecular mechanisms underlying GC and identifying novel biomarkers is crucial for improving prognostic prediction, enhancing diagnostic accuracy, and refining therapeutic strategies (8).

Emerging research underscores the pivotal roles of the tumor microenvironment (TME) in various oncogenic processes, including tumor initiation, progression, metastasis, and therapeutic response. The TME consists of a complex network of cellular and non-cellular components surrounding malignant cells, including vascular structures, diverse immune cell populations, cancer-associated fibroblasts, inflammatory mediators, extracellular matrix (ECM) proteins, and molecular signaling factors (9, 10). It plays a pivotal role in tumor growth, spread, and metastasis (11, 12), and through interactions with tumor cells, the TME can modulate the tumor’s biological behavior and response to treatment (11, 12). Within the TME, parthanatos (PA)—a distinct form of regulated cell death pertinent to cancer biology—can specifically eliminate tumor cells with accumulated DNA damage or immunosuppressive stromal cells by triggering Poly(ADP-ribose) polymerase 1 (PARP-1) overactivation and subsequent nuclear translocation of Apoptosis Inducing Factor Mitochondria associated 1(AIFM1) (13). Previous studies have shown that PA regulates the survival and death of tumor cells, particularly in the context of therapies like chemotherapy and radiotherapy (13). Under glucose-deficient conditions, PA promotes the death of gastric cancer cells by increasing intracellular oxidative stress (reactive oxygen species generation) (14). Furthermore, the role of PA in gastric cancer is not limited to regulating cell death; it is also closely associated with immune responses and metabolic characteristics within the tumor microenvironment (15). These findings suggest that PA may influence the effectiveness of cancer therapy by affecting tumor cell sensitivity to treatment (13). Therefore, conducting a comprehensive integrated analysis of both TME and PA allows for the simultaneous linking of PA-mediated cell death effects with the status of TME. This enables a more accurate assessment of tumor progression and treatment sensitivity, thereby offering a more robust biological basis for prognostic evaluation in patients.

This study innovatively integrated parthanatos (PA)-related genes with tumor microenvironment (TME) analysis in gastric cancer. Using bioinformatics and multi-algorithm screening, it identified and validated a novel prognostic gene signature, revealing its close association with immune infiltration and potential immunotherapy response, thereby providing new insights for GC prognosis and treatment.

## 2 Materials and methods

### 2.1 Data source

This study utilized publicly available TCGA RNA-seq data (375 tumor and 32 normal samples) as the training set, sourced from the official database (https://www.cancer.gov/tcga). This cohort included clinical data (age, gender, T stage, N stage, and M stage) along with survival information for 350 patients with GC. Additionally, the GSE66229 dataset, available through the Gene Expression Omnibus (GEO) database (https://www.ncbi.nlm.nih.gov/gds) and sequenced by microarray on the GPL570 platform, was downloaded. A total of 300 GC tumor tissue samples were selected from this dataset to form the validation cohort. In this study, 3,569 TME-RGs were obtained from two publicly available databases: the Molecular Signatures Database (MSigDB) (http://www.gsea-msigdb.org/gsea/msigdb/index.jsp) and Reactome Pathway (https://reactome.org/PathwayBrowser/). Furthermore, 10 PA-RGs were sourced from previous literature (16), specifically PARP-1, PAEP, MIF, AIFM1, HSP70, PAAN, ARH3, RNF146, ADPRHL2, and OGG1. The flowchart for this study was shown in **Supplementary Figure 1**.

### 2.2 Construction of differential expression analysis

In the training cohort, differential expression analysis was performed using the DESeq2 package (v 1.34.0) (17), identifying differentially expressed genes (DEGs) between tumor and control samples. A stringent threshold of |log2Fold Change (FC)| > 1 and *P* < 0.05 was applied, and these genes were designated as DEGs1. A volcano plot was generated using the ggplot2 package (v 3.4.1) (18) to visualize the distribution of DEGs1, highlighting the top 10 upregulated and downregulated genes in GC samples based on their log2FC values. Additionally, a heatmap was created with the pheatmap package (v 1.0.12) (19) to visually represent gene expression patterns. Further analysis of the expression profiles of the 10 PA-RGs in the training cohort was conducted using the single-sample gene set enrichment analysis (ssGSEA) function from the GSVA package (v 1.42.0) (20) to calculate the enrichment scores of PA-RGs across GC samples. Samples were stratified into high and low PA-RG score groups based on the optimal cutoff point, and differences in PA-RG scores between groups were assessed (*P* < 0.05). Kaplan-Meier (K-M) survival curves were generated using the survminer package (v 0.4.9) (21) to compare overall survival (OS) between the two groups (*P* < 0.05). To further explore the molecular features associated with PA-RGs scores, differential expression analysis was repeated in the training cohort to identify DEGs between the PA-RG score groups, using the same criteria of |log2FC| > 1.2 and *P* < 0.05, and these genes were designated as DEGs2. Volcano plots and heatmaps were again used to visually represent these DEGs2.

### 2.3 Recognition and enrichment analysis of candidate genes

Following the aforementioned analyses, the intersection of DEGs1, DEGs2, and 3,569 TME-RGs was utilized to identify candidate genes associated with both TME and PA in GC. The VennDiagram package (v 1.7.1) (22) was employed to visualize these candidate genes. Gene Ontology (GO) and Kyoto Encyclopedia of Genes and Genomes (KEGG) enrichment analyses were then performed to explore the biological functions and pathways of the candidate genes. Enrichment was considered significant for *P* < 0.05, with analyses conducted *via* the clusterProfiler package (v 4.6.0) (23). To effectively present the results, the GOplot package (v 1.0.2) (24) was used to graphically depict the top 5 most significant GO terms from each of the 3 GO domains, as well as the top 15 KEGG pathways, ranked by their statistical significance.

### 2.4 Selection of prognostic genes through 101 machine learning algorithms

Expression profiles of the candidate genes were integrated with survival data from 350 patients with GC in the training cohort. Univariate Cox regression analysis was conducted using the survival package (v 3.3-1) (21), with a threshold set at *P* < 0.05 and Hazard Ratio (HR) ≠ 1. Genes meeting these criteria were further tested for proportional hazards (PH) assumptions using the cox.zph function. Genes that passed the threshold of *P* > 0.05 were classified as survival-associated genes for subsequent analysis. A forest plot was generated using the forestplot package (v 2.0.1) (25) to visually represent the results.

Subsequently, a good predictive framework was constructed within the training and validation cohorts by integrating 10 distinct machine learning algorithms to create 101 potential algorithmic combinations. This analytical pipeline included multiple machine learning approaches, such as random survival forest (RSF), elastic net (Enet), Least Absolute Shrinkage and Selection Operator (LASSO), Ridge regression, stepwise Cox, CoxBoost, plsRcox, SuperPC, GBM, and survival-SVM. To evaluate the reliability and generalizability of the model, a rigorous 10-fold cross-validation process was implemented. Each model’s predictive accuracy was quantified using the C-index, calculated across both the training and validation cohorts. The objective was to identify algorithms consistently exhibiting a high C-index in both cohorts, indicating their robustness and predictive power. This comprehensive approach led to the identification of a set of prognostic genes for GC, along with their corresponding risk coefficients. The specific algorithm combinations and descriptions of their key parameters are provided in the **Appendix**.

### 2.5 Construction and validation of risk model

Based on the expression levels of the prognostic genes and their respective risk coefficients, a risk model was constructed following the formula:


$$risk\;score=\sum_{i=1}^{n}\left(coefi*Xi\right)$$

where Xi represents the expression level of prognostic genes, and coefi denotes the risk coefficient of the corresponding gene. Using the median risk score as the cutoff, the 350 patients with GC in the training cohort were stratified into high- and low-risk groups. The risk model was validated through multiple approaches (1): risk score distribution and survival status visualization using the survminer and ggrisk (v1.3) (26) packages (2), K-M analysis to demonstrate significant OS differences (*P* < 0.05), and (3) time-dependent ROC analysis (survivalROC v1.0.3) with AUC calculations at 1-, 3-, and 5-year intervals. AUC between 0.6 and 0.7 indicated that the prognostic model had certain predictive value (27). The same validation framework was applied to the validation cohort. Additionally, based on data from the training and validation sets, KM analysis for TNM staging was incorporated.

### 2.6 Association analysis of risk score with clinical characteristics

To deepen the understanding of the correlation between risk scores and clinical characteristics, and to assess the prognostic capabilities of risk scores in predicting clinical outcomes, clinical data (age, gender, T stage, N stage, and M stage) from 350 patients with GC with complete survival information in the training cohort were analyzed. Further validation of the GSE66229 dataset was performed. Distribution patterns of risk scores across different clinical subgroups in both high-risk and low-risk categories were delineated, providing insights into the heterogeneity of risk among patient populations. A comparative analysis was then performed to examine the variance in risk scores across distinct clinical subgroups (*P* < 0.05). This analysis identified potential disparities in the prognostic significance of risk scores across various stages and characteristics of GC.

### 2.7 Independent prognostic analysis and construction of a nomogram

The study integrated risk scores with clinical parameters from 350 patients with GC in the training cohort, all possessing complete survival data. A comprehensive statistical analysis was conducted using the survival package, which included (1): preliminary screening *via* univariate Cox regression (*P* < 0.05) (2), validation of PH assumptions (*P* > 0.05), and (3) final selection through multivariate Cox analysis (*P* < 0.05). This sequential approach identified good independent predictors of patient outcomes.

The identified prognostic factors were incorporated into a predictive nomogram developed with the rms package (v6.2-0) (28). This visual tool assigned weighted point values to each variable, with the total score (Total Points) allowing for the estimation of 1-, 3-, and 5-year survival probabilities. Notably, the scoring system demonstrated a positive correlation between higher total points and improved clinical prognosis.

The nomogram’s predictive performance was rigorously validated using two complementary methods (1): calibration analysis, comparing predicted versus observed survival outcomes, and (2) time-dependent ROC curve analysis (timeROC v0.4 (29)), which quantified discrimination accuracy at clinically relevant intervals. These validation procedures confirmed the model’s reliability in prognostic stratification. Decision curve analysis (DCA) was further performed to assess the clinical net benefit of the nomogram, gene risk score, and conventional clinical indicators across a threshold probability range of 0 to 0.6.

### 2.8 Immune landscape analysis

Further investigation focused on delineating the immune landscape within patients with GC. The CIBERSORT algorithm (v 1.03) (30) was applied to quantify the infiltration levels of 22 distinct immune cell types (31) across GC samples in the training cohort, with particular attention to comparing these levels between high-risk and low-risk categories (*P* < 0.05). To explore the relationship between immune cell infiltration and prognostic genes, the correlation function was used to assess their associations. Additionally, 47 immune checkpoints from published literature (32) were curated, and their expression levels were compared between high-risk and low-risk groups in the training cohort (*P* < 0.05). To evaluate potential responses to immunotherapy, TIDE scores for GC samples were calculated using the TIDE database (http://tide.dfci.harvard.edu/), and differences between risk groups were assessed (*P* < 0.05). This approach provided insights into the potential efficacy of immunotherapy across distinct risk stratifications.

### 2.9 Drug sensitivity analysis

To assess the correlation between risk categories and chemotherapy responsiveness, the pRRophetic package (v 0.5) (33) was employed to compute the IC50 values for 138 commonly used chemotherapeutic and molecular targeting agents in the training cohort. IC50 values reflect drug cytotoxicity or cell resistance, with lower IC50 values indicating greater drug efficacy. After calculating the IC50 values, comparisons were made between the higher-risk and lower-risk categories to identify significant differences (*P* < 0.05). The ggplot2 package was utilized to graphically display the top 5 drugs with the most pronounced differences in IC50 values through box plots.

### 2.10 Expression validation of biomarkers

Five GC tumor tissue samples and five adjacent tumor normal tissue samples were collected at Shaanxi Provincial People’s Hospital. All samples underwent quantitative real-time polymerase chain reaction (RT-qPCR) analysis, approved by the Medical Ethics Committee of Shaanxi Provincial People’s Hospital. Total RNA was extracted from approximately 50 mg of each tissue sample using TRIzol reagent (Ambion, USA) according to the manufacturer’s instructions. RNA concentration and purity were assessed using a NanoPhotometer N50, and samples with A260/A280 ratios between 1.8 and 2.0 were considered suitable for downstream applications. First-strand cDNA was synthesized from 2 µg of total RNA using the SureScript First-Strand cDNA Synthesis Kit (Servicebio, China) in a 20 µL reaction volume. The reverse transcription reaction was carried out under the following conditions: 25 °C for 5 min, 50 °C for 15 min, and 85 °C for 5 sec, followed by hold at 4 °C. RT-qPCR was performed using 2× Universal Blue SYBR Green qPCR Master Mix (Servicebio, China) on a CFX Connect Real-Time PCR System (Bio-Rad, USA). Each 10 µL reaction contained 3 µL of diluted cDNA, 5 µL of master mix, and 0.5 µM each of forward and reverse primers. The amplification protocol consisted of an initial denaturation at 95 °C for 1 min, followed by 40 cycles of 95 °C for 20 sec, 55 °C for 20 sec, and 72 °C for 30 sec. Melting curve analysis was performed to confirm primer specificity. Primer sequences (**Supplementary Table 1**) and GAPDH (internal control) were used for amplification. Relative gene expression was quantified using the 2^−ΔΔCt^ method (34).

### 2.11 Immunohistochemistry

GC tumor tissue samples and adjacent tumor normal tissue samples were collected at Shaanxi Provincial People’s Hospital. Paraffin sections of gastric cancer tissues and adjacent tumor normal tissues were dewaxed, followed by antigen retrieval for 15 minutes. Endogenous peroxidase was blocked with 3% hydrogen peroxide, and non-specific binding sites were blocked with normal goat serum. Subsequently, primary antibodies against CD36 (18836-1-AP, Sanying, China) or KIT (AF6153, Affinity, USA) were added, and the sections were incubated overnight at 4°C followed by incubation with HRP-conjugated secondary antibody (K5007, Dako, Denmark) at 37 °C for 30 minutes. After DAB staining and hematoxylin counterstaining, the sections were sequentially dehydrated, cleared, mounted with neutral balsam, and observed under a microscope. The experiment was repeated 3 times.

### 2.12 Immunofluorescence staining of tissue

Paraffin sections of gastric cancer tissues and adjacent tumor normal tissues were dewaxed, followed by antigen retrieval for 15 minutes. Endogenous peroxidase was blocked with 3% hydrogen peroxide, and non-specific binding sites were blocked with normal goat serum. Subsequently, primary antibodies against CD3 (16-0032-81, invitrogen, China), CD4 (67786-1-Ig, Sanying, China), FOXP3 (98377, CST, China) or CD68 (ab213363, abcam, UK), CD86(bs-1035R, bioss, USA), CD206 (18704-1-AP, Sanying, China) or *AKAP12*(ab198895, abcam, UK), CD14(ab133335, abcam, UK) were applied, and the sections were incubated overnight at 4°C. After returning to room temperature, fluorescent secondary antibodies were added and incubated at 37°C for 30 minutes in the dark. Nuclei were stained with DAPI. Finally, the sections were mounted with an anti-fade mounting medium and imaged under a fluorescence microscope. The experiment was repeated 3 times.

### 2.13 CCK8 assay

Cell proliferation was measured using the CCK-8 assay kit (APExBIO, Houston, USA). The MKN45 gastric cancer cell line was kindly provided by the Shaanxi Provincial People’s Hospital. MKN-45 cells were inoculated into 96-well cell culture plates at a density of 3×10^3/^well and cultured overnight at 37°C in a 5% CO2 incubator. The cells were then treated with *CD36* inhibitor (SMS121, HY-163541, MCE, USA), *KIT* inhibitor (c-Kit-IN-5-1, HY-18302, MCE, USA).10ul of CCK8 was added to each well and cultured at 37°C for 2h, the enzyme marker (BioTek, Shanghai, China) was used to determine the absorbance (A450nm) values. Cell viability (%) was calculated using the formula: 100×(experimental absorbance)/(control absorbance). The experiment was repeated 3 times.

### 2.14 Transwell assay

Cell migration was measured using the Transwell assay kit (353097, FALCON, USA). After MKN45 cells were treated with *CD36/KIT* inhibitors, the cells were digested with trypsin, cells were adjusted to a concentration of 3×10^5^ cells/ml. Subsequently, the cell suspension was added to Transwell inserts and cultured in a 37°C, 5% CO_2_ incubator for 24 h. Following incubation, cells were fixed with 70% ice-cold ethanol for 1 h, then stained with 0.5% crystal violet staining solution at room temperature. Finally, cells were observed and photographed under a microscope, and the number of migrated cells was counted and the experiment was repeated 3 times.

### 2.15 Western blotting

Proteins were extracted from MKN45 cells treated with *CD36/KIT* inhibitors. The protein concentration was determined and standardized by the BCA method. Equal amounts of protein samples were separated by SDS-PAGE gel electrophoresis and then transferred onto PVDF membranes. The membranes were blocked with 5% non-fat milk on a shaker at room temperature for 2 h, followed by incubation with primary antibodies overnight at 4°C: mouse monoclonal antibody GAPDH (36KD, 60004-1-Ig, Sanying, China); rabbit polyclonal antibody CD36 (88KD, 18836-1-AP, Sanying, China); rabbit polyclonal antibody KIT (110KD, AF6153, Affinity, USA). On the next day, the membranes were washed 3 times, incubated with HRP-conjugated secondary antibodies at room temperature for 1 h, and then washed 3 times. ECL chemiluminescence kit was used for development, the gray value of the target proteins was quantitatively analyzed, and the experiment was repeated 3 times.

### 2.16 Enzyme-linked immunosorbent assay

The secretion levels of cytokines were detected using the Human IFN-γ ELISA Kit (USEA049Hu, Yunkelong, China), Human TNF-α ELISA Kit (USEA133Hu, Yunkelong, China) and Human IL-10 ELISA Kit (USEA056Hu, Yunkelong, China). Supernatants were collected from the culture medium of MKN45 cells treated with *CD36/KIT* inhibitors after centrifugation, the experiment was performed following the manufacturer’s instructions of the ELISA kit. Standard substances were serially diluted and added to the pre-coated microplate together with the samples, followed by incubation at 37°C for 1 h. The liquid was discarded and the microplate was washed with washing buffer. Detection Solution A was added and incubated at 37°C for 1 h; after washing, Detection Solution B was added and incubated at room temperature for 30 min in the dark, followed by thorough washing again. Chromogenic substrate was added for color development in the dark for 20 min, and the reaction was terminated with stop solution. The absorbance was immediately measured at 450 nm using a microplate reader, and the target protein concentration in samples was calculated according to the standard curve. The experiment was repeated three times.

### 2.17 Immunofluorescence staining of cell climbing sheets

MKN45 cells were seeded on cell climbing sheets and cultured until adherent. After treatment with *CD36/KIT* inhibitors, the cells were fixed with 4% paraformaldehyde at room temperature for 15 min, permeabilized with 0.1% Triton X-100 for 5 min, and rinsed three times. The cells were blocked with normal goat serum at room temperature for 30 min, followed by the addition of primary antibodies: rabbit polyclonal antibody PARP-1 (DF7198, Affinity, USA) and rabbit polyclonal antibody AIFM1 (DF7021, Affinity, USA), and incubated overnight at 4°C. On the next day, after rinsing, secondary antibody: CY3-conjugated goat anti-rabbit IgG (BA1032, BOSTER, USA) was added, and the cells were incubated in the dark at 37°C for 1 h. The nuclei were stained with DAPI in the dark for 5 min after rinsing. After thorough rinsing, the cells were mounted with anti-fluorescence quenching mounting medium, observed and imaged under a fluorescence microscope, and the experiment was repeated 3 times.

### 2.18 Statistical analysis

All statistical analyses were conducted using R software (version 4.2.2; R Foundation for Statistical Computing, Vienna, Austria). Specifically, the clusterProfiler package was used for GO and KEGG enrichment analysis, the DESeq2 package for gene differential expression analysis, the glmnet package for constructing Lasso-Logistic regression model, the rms package for plotting nomogram, calibration curves, and decision curves, and the pROC package for ROC analysis. All assays were performed in triplicate, and the data are expressed as the mean ± standard deviation (SD). Statistical analysis was performed using GraphPad Prism 25.0((Version 25.0, San Diego, CA, USA).). Statistical analyses were conducted using GraphPad Prism software Differences between two groups were evaluated by Student’s t-test or the Wilcoxon rank-sum test (Mann-Whitney U test), while one-way analysis of variance (ANOVA) was applied for comparisons among multiple groups. All data were expressed as mean ± SD, where *P < 0.05, **P < 0.01, ***P < 0.001, and ****P < 0.0001 were considered statistically significant A two-sided *P* < 0.05 was considered statistically significant.

## 3 Results

### 3.1 Prognostic significance of PA-RGs scoring in GC

**Figure 1:**
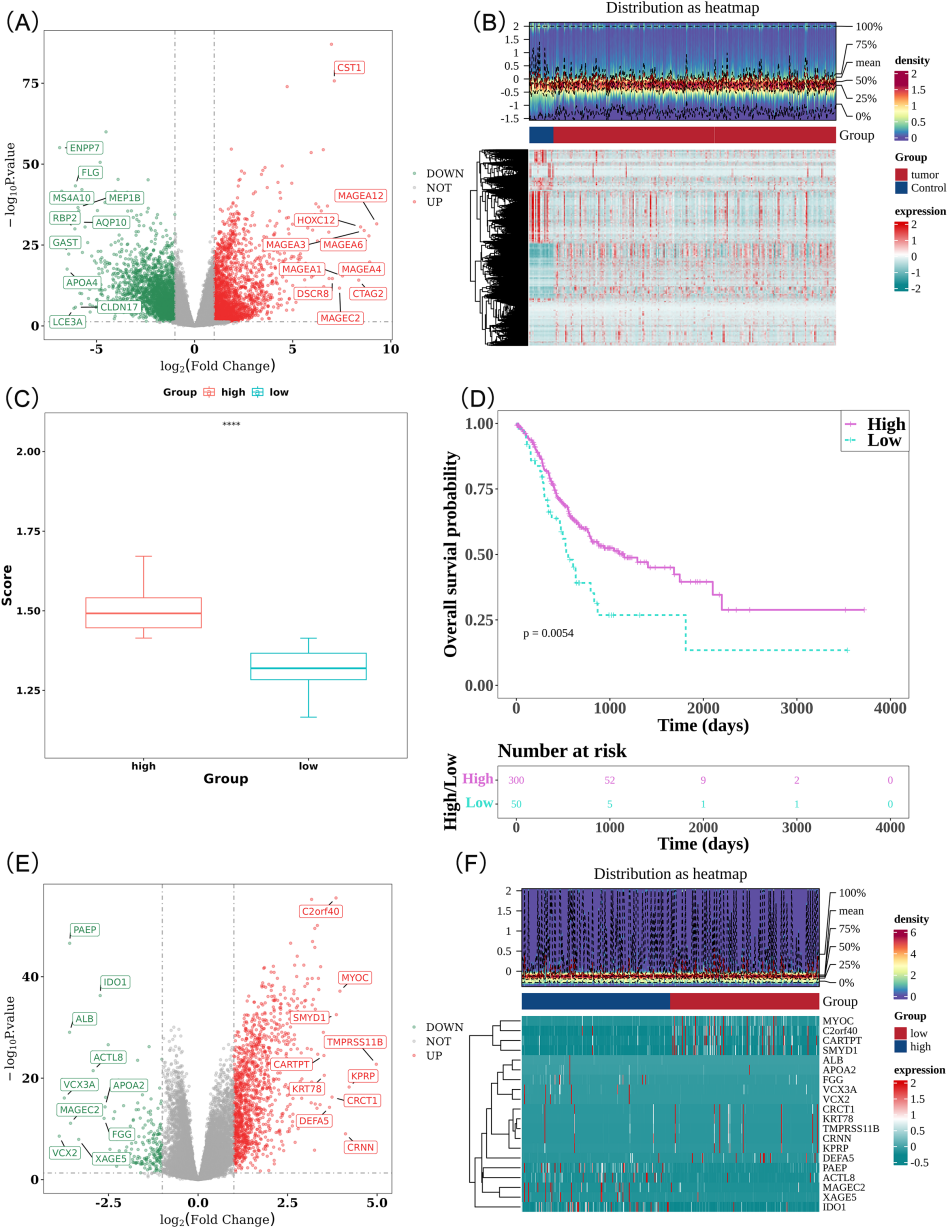
Analysis of differentially expressed genes and prognostic significance of PA-RGs scoring in GC. **(A)** Volcano plot of differentially expressed genes between gastric adenocarcinoma and normal samples. Red indicates genes upregulated in gastric adenocarcinoma, while green indicates genes downregulated in gastric adenocarcinoma samples. **(B)** Heatmap of differentially expressed genes between gastric adenocarcinoma and normal samples. The upper half of the figure shows the density heatmap of data distribution, while the lower half shows the expression level heatmap. **(C)** Differences in ssGSEA scores of PA-RGs. **(D)** Kaplan-Meier survival curves for patients in high- and low-PA-RGs score groups. **(E)** Volcano plot of differentially expressed genes between high- and low-scoring PA-RGs groups. Red indicates genes upregulated in the high-PA-RGs score group, while green indicates genes downregulated in the high-PA-RGs score group. **(F)** Heatmap of differentially expressed genes between high- and low-scoring PA-RG groups. The upper half of the figure shows the density heatmap of data distribution, while the lower half shows the expression level heatmap.

In the training cohort, differential expression analysis between tumor and control samples revealed 4,570 DEGs, comprising 2,208 upregulated and 2,362 downregulated genes in GC (**Figures 1A, B**). PA-RG scores were then calculated for GC samples, which were stratified into high and low score groups. The high PA-RG score group exhibited significantly higher scores (*P* < 0.0001, **Figure 1C**), while K-M analysis indicated worse OS in the low score group (*P* = 0.0054, **Figure 1D**), suggesting the potential of PA-RGs as prognostic markers in GC. Additionally, comparisons between PA-RG score groups revealed 1,417 DEGs (1,230 upregulated and 187 downregulated, **Figures 1E, F**).

### 3.2 Selection of candidate genes and their enriched associated pathways

**Figure 2:**
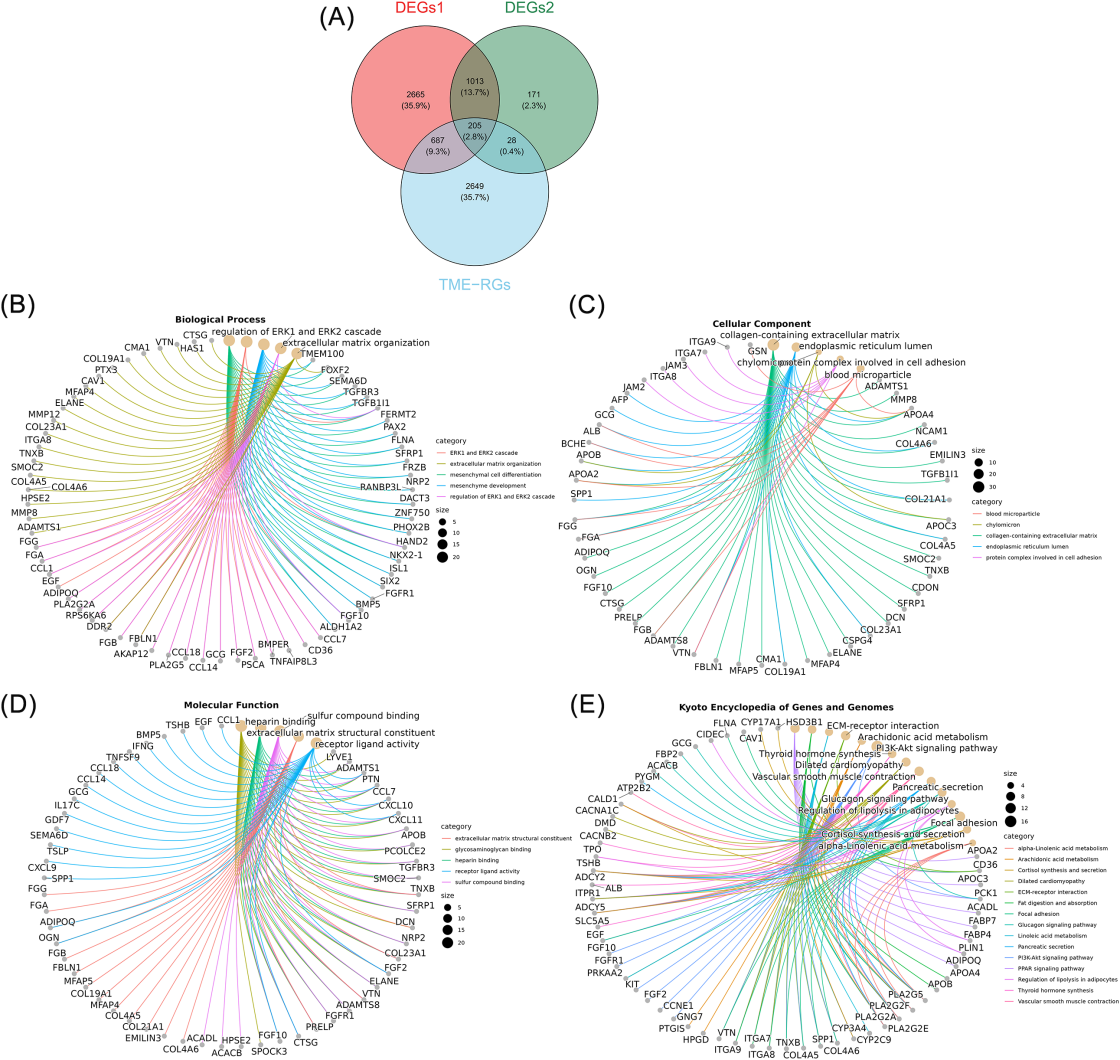
Selection and enrichment of candidate genes. **(A)** Venn diagram depicting the intersection of DEGs1, DEGs2, and TME-RGs. **(B–D)** GO functional enrichment of candidate genes. **(B–D)** show the enrichment results for BP, CC, and MF, respectively. **(E)** KEGG pathway enrichment of candidate genes.

By intersecting 4,570 DEGs1, 1,417 DEGs2, and 3,569 TME-RGs, 205 candidate genes associated with both TME and PA in GC were identified (**Figure 2A**). These 205 genes were subjected to enrichment analysis, resulting in 990 GO terms, including 878 biological processes (BP), 40 cellular components (CC), and 72 molecular functions (MF). Key biological processes involved the ERK1/2 signaling cascade, ECM remodeling, mesenchymal differentiation, mesenchyme development, collagen-rich ECM, cell adhesion-associated protein complexes, heparin binding, and glycosaminoglycan interactions, all of which contribute to GC pathogenesis (**Figures 2B–D**). Furthermore, the candidate genes were enriched in 36 KEGG pathways, such as arachidonic acid metabolism, ECM-receptor crosstalk, PI3K-Akt signaling, focal adhesion dynamics, PPAR signaling, and adipocyte lipolysis regulation (**Figure 2E**), all of which are likely involved in tumor cell proliferation, invasion, metastatic potential, and the establishment of a pro-inflammatory TME in GC.

### 3.3 Identification of prognostic genes: *EGF*, *PCOLCE2*, *CD36*, *ADAMTS8*, *CIDEC*, *KIT*, and *AKAP12*

**Figure 3:**
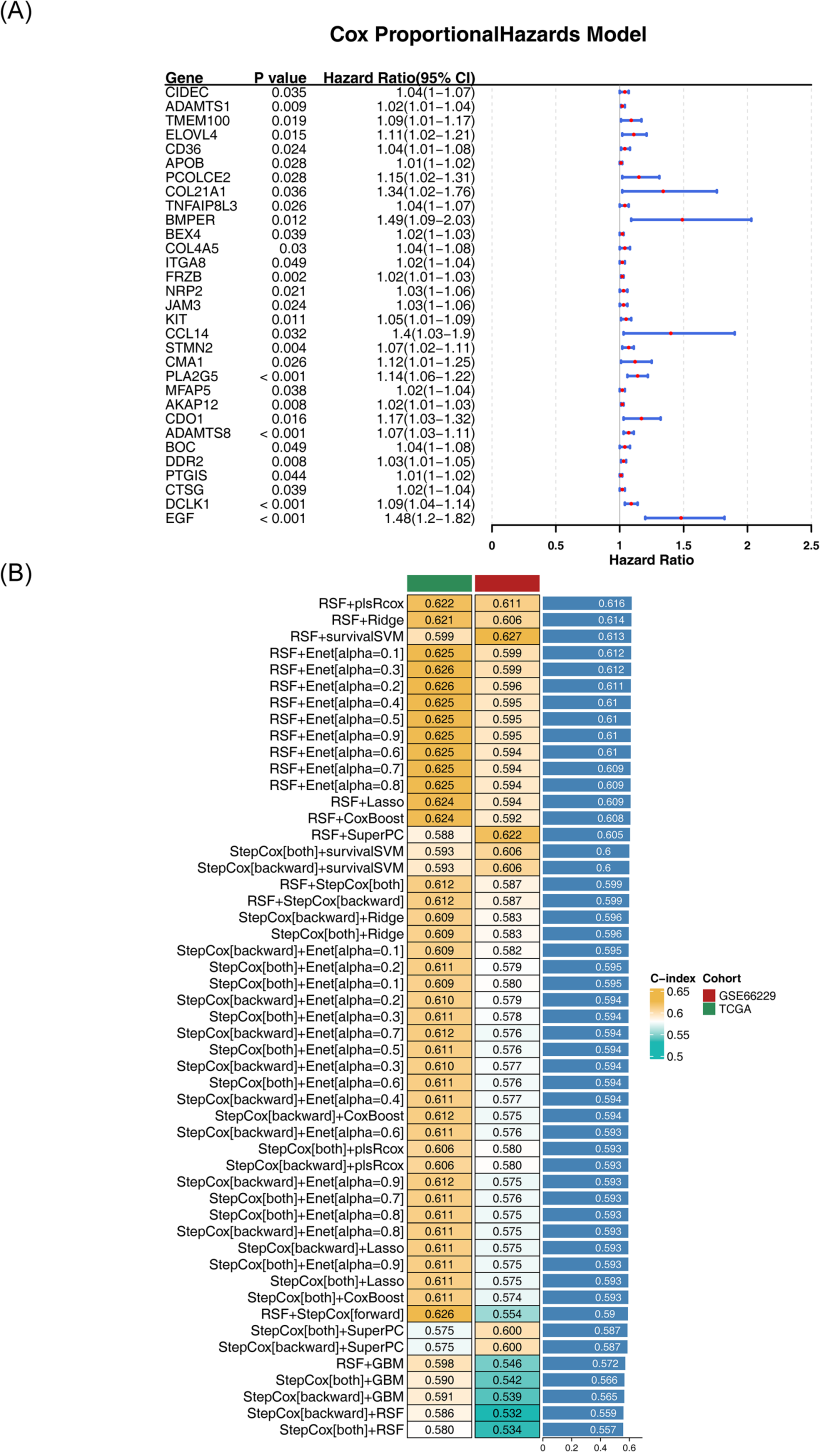
Machine learning and the development of prognostic models. **(A)** Forest plot of univariate Cox regression analysis. The Hazard Ratio (HR) in the figure represents the ratio of hazard function values. When HR > 1, the exposure factor promotes the occurrence of positive events; when HR< 1, the exposure factor inhibits the occurrence of positive events; when HR = 1, the exposure factor has no effect on the occurrence of positive events. **(B)** Analysis of 101 permutations and combinations of ten machine learning algorithms. The left column lists the machine learning algorithms, the middle column shows the corresponding C-index values for the training and validation sets, and the right column displays the overall C-index.

A univariate Cox regression analysis was performed on the 205 candidate genes within the training cohort, identifying 31 genes that met the established criteria (*P* < 0.05 and HR ≠ 1) (**Figure 3A**). All 31 genes passed the PH assumption test (*P* > 0.05) (**Supplementary Figure 2**) and were thus classified as survival-associated genes for further selection in machine learning algorithms.

Subsequent analyses were conducted in both the training and validation cohorts, utilizing 10 machine learning algorithms to generate 101 algorithmic combinations, incorporating survival-associated genes for further refinement. Of particular note was the RSF + plsRcox algorithm, which achieved a C-index of 0.622 in the training cohort and 0.611 in the validation cohort, exceeding the critical threshold of 0.6 in both instances (**Figure 3B**). The C-index reflects the model’s ability to distinguish between patients with different prognoses. When the C-index is between 0.6 and 0.7, it indicates that the model can somewhat differentiate between patients with better and worse prognoses, although its discriminatory power is moderate (35). This suggests that the model has potential clinical application value. Given its good performance, this model was selected for generating risk coefficients and identifying the 7 prognostic genes integral to the model’s framework: *EGF*, *PCOLCE2*, *CD36*, *ADAMTS8*, *CIDEC*, *KIT*, and *AKAP12*.

### 3.4 Good predictive ability of the risk model

**Figure 4:**
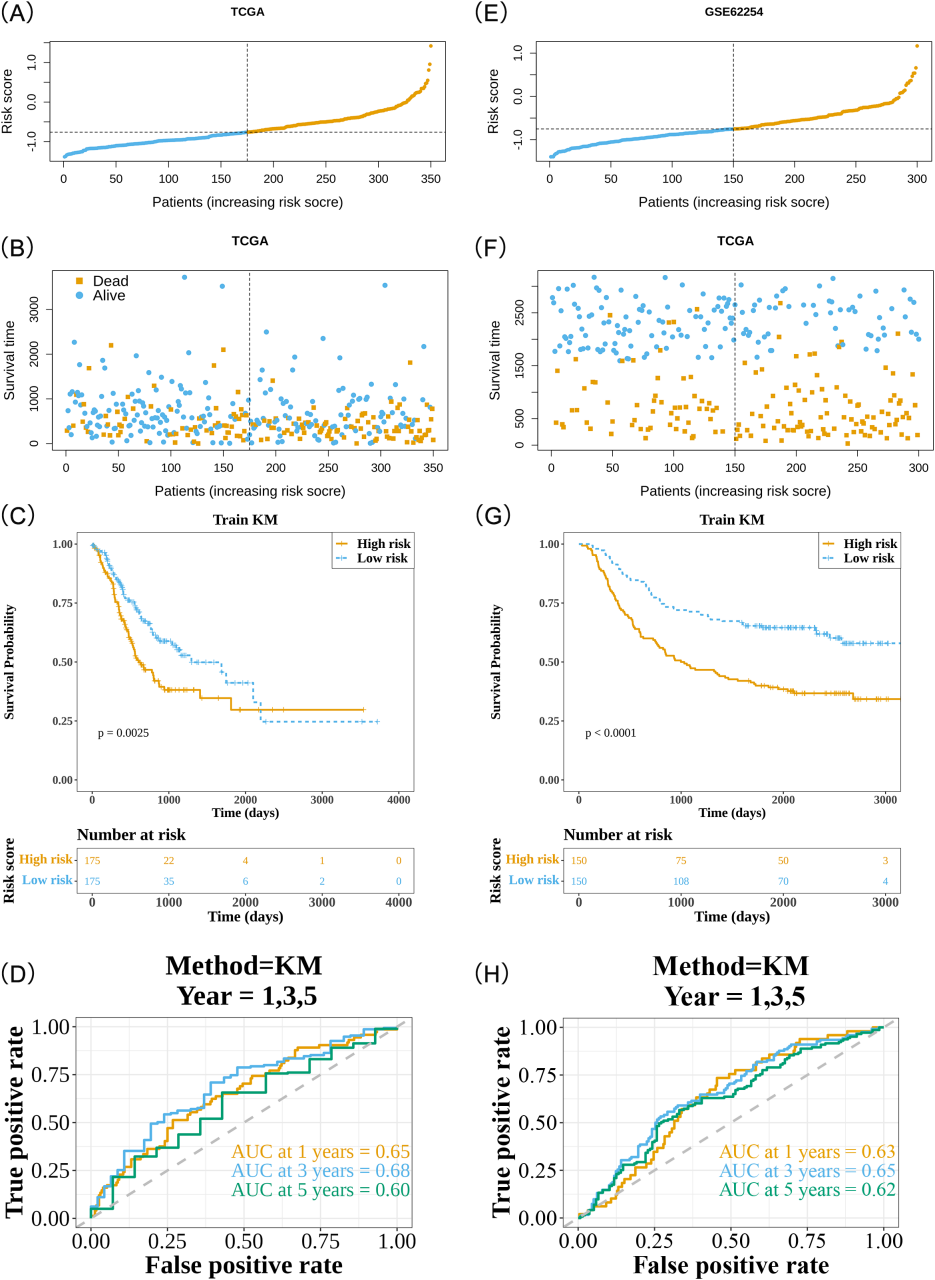
Evaluation and validation of the risk model. **(A)** Risk curve for high- and low-risk groups of patients with GC in the training cohort. The x-axis represents the risk score, with patients’ risk scores arranged from left to right. Orange dots denote high-risk patients, and blue dots indicate low-risk patients. **(B)** Scatter plot of high- and low-risk groups for patients with GC in the training cohort. Orange dots represent deceased patients, while blue dots represent survivors. **(C)** Kaplan-Meier survival analysis of prognostic genes in the training cohort. The x-axis indicates overall survival time (days), and the y-axis represents survival probability. Orange curves represent the high-risk group, while blue curves represent the low-risk group. **(D)** Time-dependent ROC curves for 1-, 3-, and 5-year survival predictions in patients with GC from the training cohort. The x-axis represents specificity, the y-axis represents sensitivity, and the area under the curve (AUC) is enclosed by the curve and the x-axis. A higher AUC value indicates superior diagnostic performance. **(E)** Risk curve of high- and low-risk groups for GC patients in the validation cohort. The x-axis indicates the risk score, with patients’ risk scores ascending from left to right; orange dots represent high-risk patients, and blue dots denote low-risk patients. **(F)** Scatter plot of high- and low-risk groups for GC patients in the validation cohort. Orange dots indicate deceased patients, and blue dots represent surviving patients. **(G)** Kaplan-Meier survival analysis of prognostic genes in the validation cohort. X-axis indicates overall survival time (days), Y-axis represents survival probability; orange curves indicate high-risk group, blue curves correspond to low-risk group. **(H)** Time-dependent ROC curves for 1-, 3-, and 5-year survival in GC patients of the validation cohort. The x-axis represents specificity, the y-axis indicates sensitivity, and the area enclosed by the curve and the x-axis is termed AUC.

A risk model was developed using the expression profiles of prognostic genes and their associated risk coefficients. In the training cohort, 350 samples with survival data were stratified into two distinct groups: 175 higher-risk and 175 lower-risk categories. The risk curve showed a clear increase in mortality as the risk score rose (**Figures 4A, B**). As expected, the higher-risk group exhibited significantly poorer OS compared to the lower-risk group (*P* = 0.0025) (**Figure 4C**). KM analysis of TNM staging in the training cohort revealed no significant differences between T stages, whereas significant differences were observed between N and M stages (N: *P* = 0.0012, M: *P* = 0.012) (**Supplementary Figure 3A**). The ROC curve further validated the model’s predictive accuracy, with AUC values of 0.65, 0.68, and 0.60 for 1, 3, and 5-year predictions, respectively (**Figure 4D**).

This predictive efficacy was corroborated in the validation cohort, which included 300 samples with survival data, divided into 150 higher-risk and 150 lower-risk categories (**Figures 4E, F**). Similar to the training cohort, the high-risk group was associated with worse OS (*P* < 0.0001) (**Figure 4G**). In the KM analysis of the validation cohort, significant differences were observed across TNM stages (T: *P* < 0.0001, N: *P* < 0.0001, M: *P* < 0.0001) (**Supplementary Figure 3B**). The AUC values for 1, 3, and 5-year predictions in the validation cohort were 0.63, 0.65, and 0.62, respectively (**Figure 4H**). These results underscore the reliability of the model and its potential clinical utility in prognostic assessment for patients with GC.

### 3.5 Revealing the distribution of clinical subgroups in higher-risk and lower-risk categories

**Figure 5:**
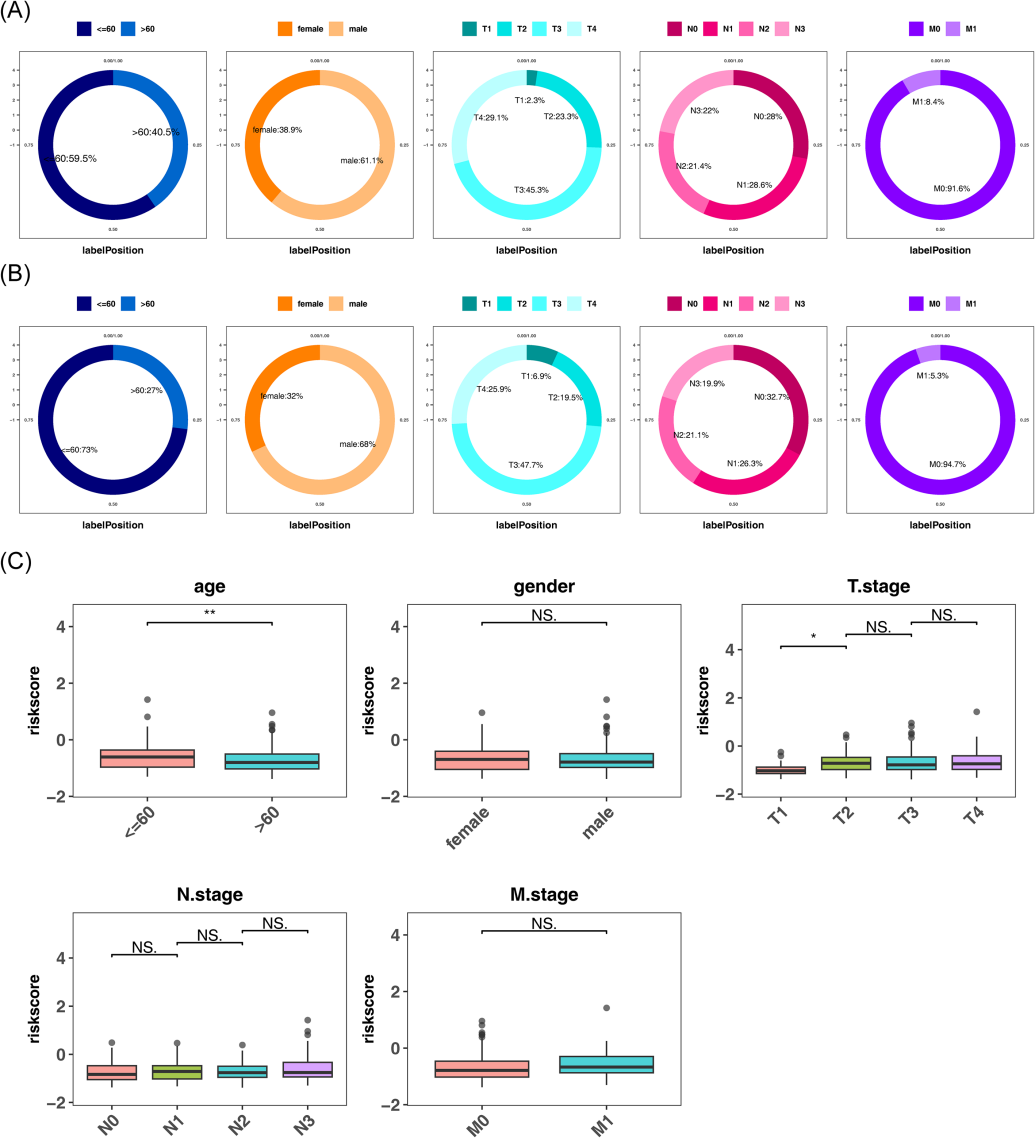
Association analysis between risk scores and clinical characteristics. **(A, B)** Distribution of patients in high- and low-risk groups across clinical subgroups. Panel A shows the distribution for the high-risk group, and **(B)** for the low-risk group. From left to right, the panels display the percentage distributions across different categories: Age, Gender, T-stage, N-stage, and M-stage. **(C)** Differences in risk scores across clinical subgroups. NS indicates no significant difference between groups; * denotes *P* < 0.05; ** denotes *P* < 0.01.

The distribution patterns of different clinical subgroups within the higher-risk and lower-risk categories were visualized in the training set, including age, gender, T stage, N stage, and M stage. In the higher-risk category, 40.5% were over 60 years old, and 59.5% were 60 or younger; 38.9% were female, and 61.1% were male; T stage distributions were 2.3% for T1, 23.3% for T2, 45.3% for T3, and 29.1% for T4; N stage distributions were 28% for N0, 28.6% for N1, 21.4% for N2, and 22% for N3; M stage distributions were 91.6% for M0 and 8.4% for M1 (**Figure 5A**). In the lower-risk category, 27% were over 60 years old, and 73% were 60 or younger; 32% were female, and 68% were male; T stage distributions were 6.9% for T1, 19.5% for T2, 47.7% for T3, and 25.9% for T4; N stage distributions were 32.7% for N0, 26.3% for N1, 21.1% for N2, and 19.9% for N3; M stage distributions were 94.7% for M0 and 5.3% for M1 (**Figure 5B**). These findings suggest that older male patients may be at higher risk of disease. In terms of TNM staging, the higher-risk category had a larger proportion in later T stages (T3 and T4) and N stages (N2 and N3), while the lower-risk category had higher proportions in earlier T stages (T1 and T2) and N stages (N0 and N1). The higher-risk category also had a greater proportion of distant metastasis (M1), indicating a strong association between distant metastasis and increased disease risk. These results highlight that TNM staging is closely related to disease risk, prompting further investigation of risk score variations across these clinical subtypes. Notably, significant differences in risk scores were observed based on age and T stage (*P* < 0.05) (**Figure 5C**). In the validation set, clinical subgroup analyses were also conducted for high- and low-risk groups. Results indicated consistent age and gender distributions across both risk groups, with similar TNM staging distributions. Compared to the training set, although T1 stage was absent in T staging, outcomes for other stages largely aligned with those observed in the training set (**Supplementary Figure 4A**). This further validated the conclusions drawn from the training set. Additionally, significant differences in the risk scores were observed based on age, T stage, and N stage (*P* < 0.05) (**Supplementary Figure 4B**). These observations suggest that risk scores correlate with clinical characteristics and cancer staging in patients with GC, with more advanced stages likely associated with higher risk scores. Patients in the high-risk category may require more intensive monitoring and potentially more aggressive treatment, while those in the low-risk category may have a better prognosis.

### 3.6 Developing a nomogram with good predictive performance

**Figure 6:**
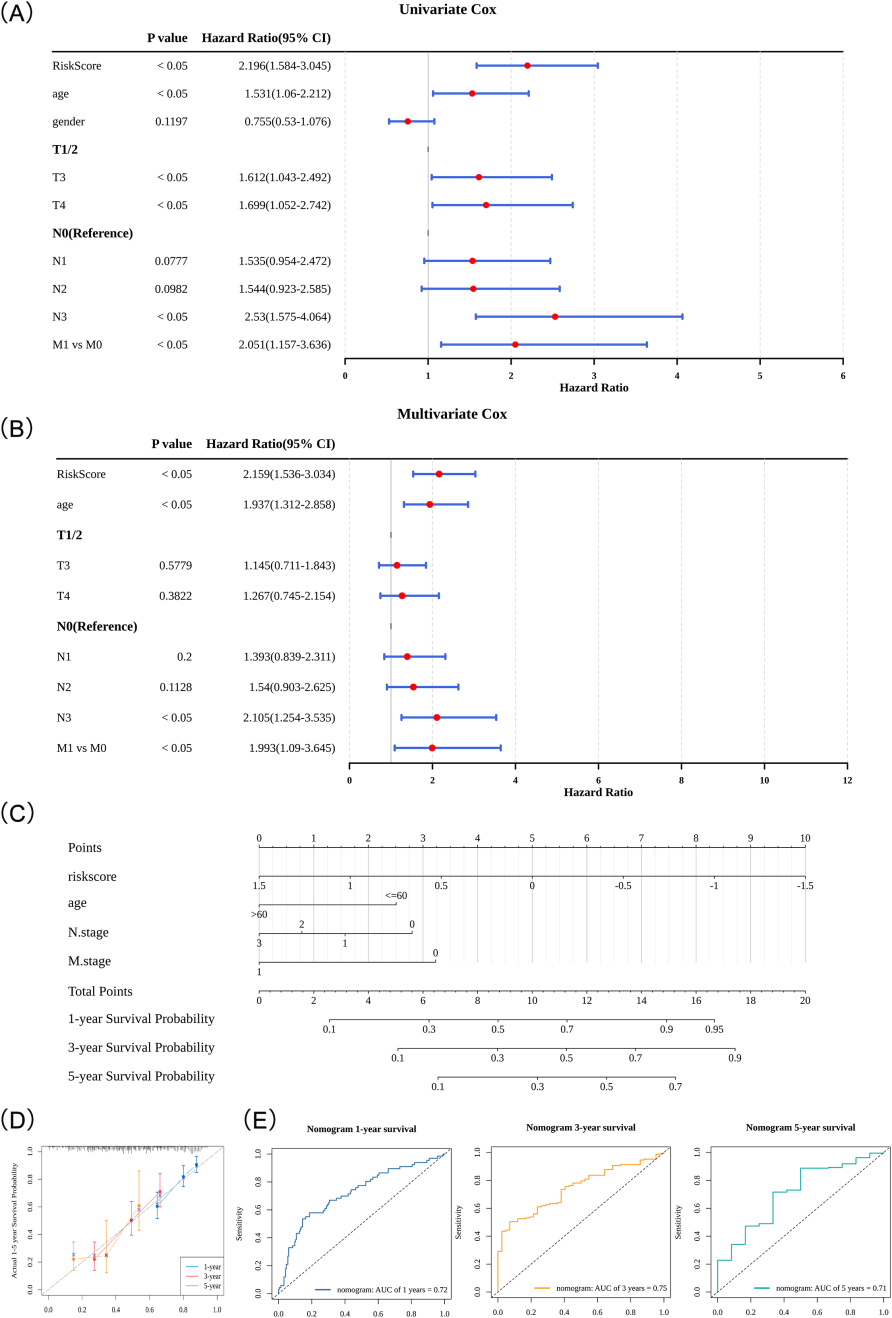
Independent prognostic analysis and nomogram construction. **(A)** Results of univariate Cox analysis. The leftmost column lists risk scores and clinical characteristics, followed by the corresponding *P*-values and hazard ratios (HR) with 95% confidence intervals (in parentheses). In the right plot, red dots represent HR values, and the flanking line segments represent the 95% confidence intervals. **(B)** Results of multivariate Cox analysis. **(C)** Nomogram predicting 1-, 3-, and 5-year survival probabilities for GC patients. Part 1 (Points): Scores are assigned based on specific values of risk factors. Part 2 (Variables): Horizontal scales represent the total contribution of each variable to the outcome, with tick marks denoting variable values. Part 3 (Total Points): The sum of individual scores from all variables. Part 4 (Predicted Survival Probabilities): Converts total points into 1-, 3-, and 5-year survival probabilities. **(D)** 1-, 3-, and 5-year calibration curves for the nomogram. The x-axis represents predicted event rates, and the y-axis represents observed event rates, with both ranging from 0 to 1. **(E)** 1-, 3-, and 5-year ROC curves for the nomogram.

A series of analyses were performed by integrating risk scores with clinical characteristics of patients with GC, ultimately identifying risk scores, age, N stage, and M stage as independent prognostic factors for GC (*P* < 0.05) (**Figures 6A, B**; **Supplementary Figure 5**). To leverage the prognostic implications of these factors, a comprehensive nomogram was developed to refine clinical prognostication for patients with GC (**Figure 6C**). This tool calculated the 1-, 3-, and 5-year survival probabilities for patients with GC, assigning a weight to each factor’s contribution to the outcome. Calibration curves demonstrated a strong alignment between predicted survival probabilities and the reference line, highlighting the high predictive accuracy of the nomogram (**Figure 6D**). ROC curves further confirmed the nomogram’s superior predictive performance, with AUC values of 0.72, 0.75, and 0.71 for 1-, 3-, and 5-year predictions, respectively (**Figure 6E**), these values notably outperformed those obtained using risk scores alone. In addition, the DCA results demonstrated that the nomogram established in this study yielded a sustained positive net benefit within the clinically common threshold range of 0 to 0.6, and its clinical decision-making value was significantly superior to that of the single genetic risk score and TNM staging-related indicators (**Supplementary Figure 6**), further validating the favorable predictive performance of this nomogram.

### 3.7 Revealing the immune landscape and treatment of individuals with distinct risk profiles

**Figure 7:**
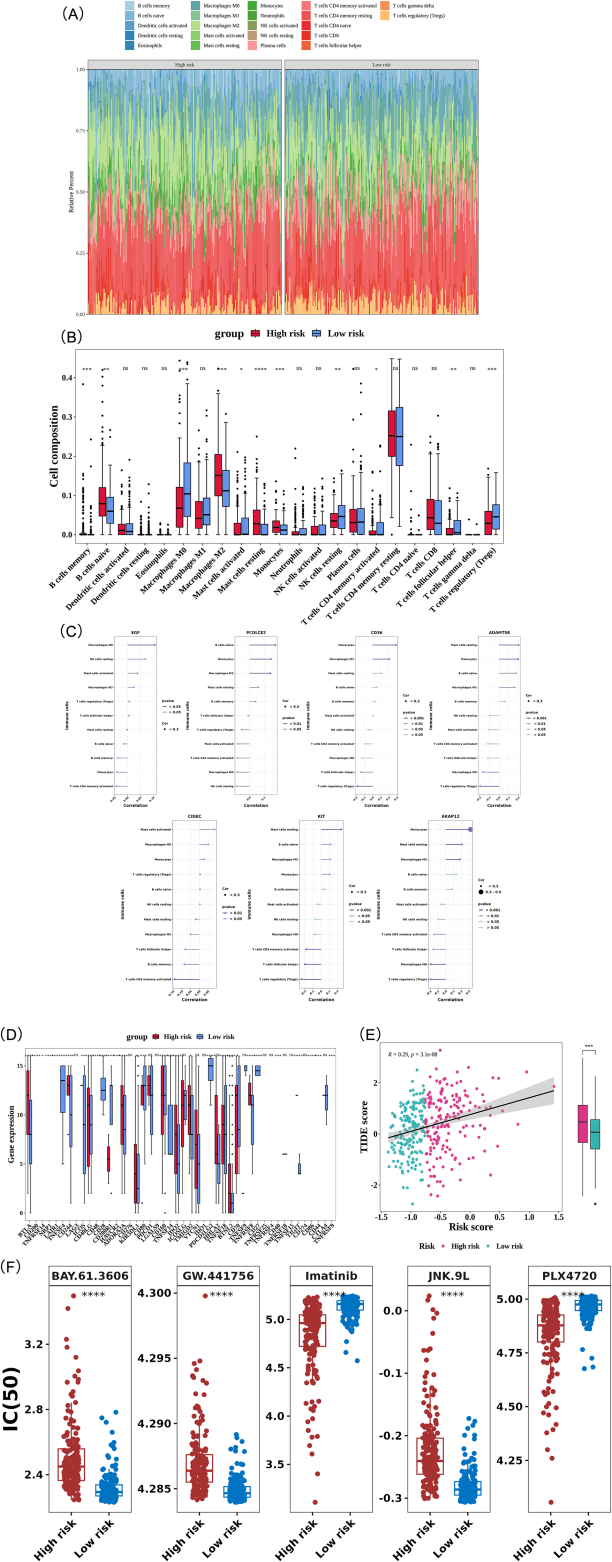
Tumor immune microenvironment analysis. **(A)** Immune cell infiltration abundance. Colors represent distinct immune cell types. **(B)** Immune cell map showing differences between high- and low-risk groups. NS indicates no significant difference between groups; * denotes *P* < 0.05; ** denotes *P* < 0.01; *** denotes *P* < 0.001; **** denotes *P* < 0.0001. **(C)** Correlation analysis between prognostic genes and differential immune cells. **(D)** Differential expression of immune checkpoints between high- and low-risk groups (box plot). The x-axis represents immune checkpoint molecules, and the y-axis represents immune scores in tumor samples. Colors: Red (high-risk group), blue (low-risk group).NS indicates no significant difference between groups; * denotes *P* < 0.05; ** denotes *P* < 0.01; *** denotes *P* < 0.001; **** denotes *P* < 0.0001. **(E)** Differences in TIDE scores between high- and low-risk groups. *** denotes *P* < 0.001. **(F)** Drug sensitivity disparities between high- and low-risk cohorts. **** denotes *P* < 0.0001.

**Figure 8:**
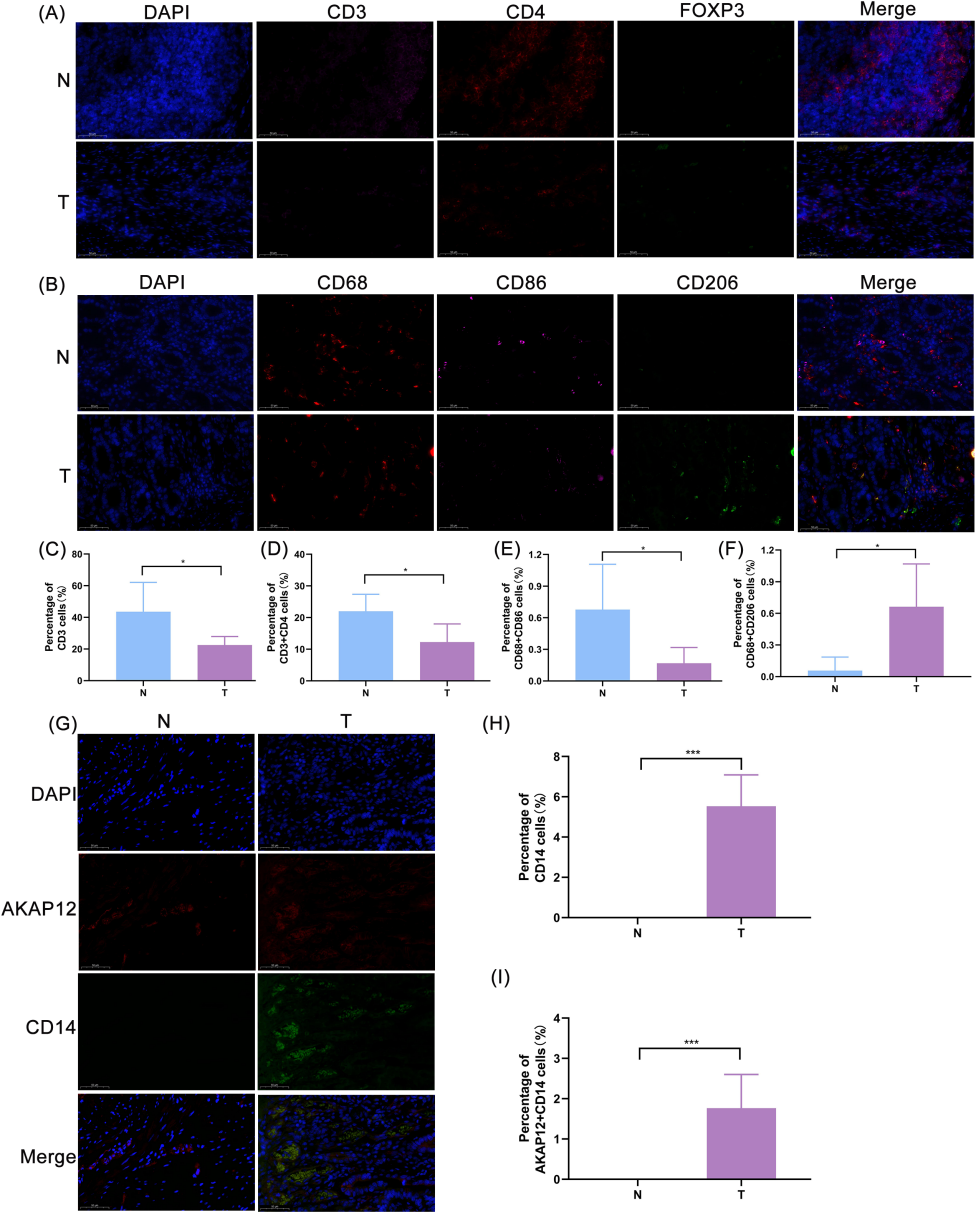
Validation of immune cell infiltration characteristics and analysis of their correlation with prognostic genes. **(A)** Immunofluorescence staining results of T cells in gastric cancer tissues and adjacent normal tissues. N: Adjacent normal, T: Tumor. Blue: DAPI (nuclear staining); Pink: CD3; Red: CD4; Green: FOXP3; Merge shows the colocalization result of the aforementioned markers. Scale bar: 50 μm. **(B)** Immunofluorescence staining results of macrophages in gastric cancer tissues and adjacent normal tissues; Blue: DAPI (nuclear staining); Red: CD68; Pink: CD86; Green: CD206; Merge shows the colocalization result of the aforementioned markers. Scale bar: 50 μm. **(C)** Differential analysis of the number of CD3^+^cells between gastric cancer tissues and adjacent normal tissues.* denotes *P* < 0.05. **(D)** Differential analysis of the number of CD3^+^CD4^+^ cells between gastric cancer tissues and adjacent normal tissues. * denotes *P* < 0.05. **(E)** Differential analysis of the number of CD68^+^CD86^+^ cells between gastric cancer tissues and adjacent normal tissues. * denotes *P* < 0.05. **(F)** Differential analysis of the number of CD68^+^CD206^+^cells between gastric cancer tissues and adjacent normal tissues. * denotes *P* < 0.05. **(G)** Co-immunofluorescence staining results of CD14^+^monocytes and *AKAP12* in gastric cancer tissues and adjacent normal tissues; Blue: DAPI (nuclear staining); Red: *AKAP12*; Green: CD14; Merge shows the colocalization result of the three markers. Scale bar: 50 μm. **(H)** Differential analysis of the number of CD14^+^cells between gastric cancer tissues and adjacent normal tissues.*** denotes *P* < 0.001. **(I)** Differential analysis of the number of AKAP12^+^CD14^+^ cells between gastric cancer tissues and adjacent normal tissues.*** denotes *P* < 0.001.

This study further evaluated the infiltration levels of 22 distinct immune cell types across the two risk categories of patients with GC (**Figure 7A**). A significant difference in the abundance of 11 immune cell types was observed between the groups (*P* < 0.05). Notably, memory B cells, naive B cells, M2 macrophages, resting mast cells, and monocytes were more abundant in the higher-risk category (*P* < 0.05), suggesting their potential role in facilitating tumor progression and suppressing immune responses. In contrast, M0 macrophages, activated mast cells, resting NK cells, activated memory CD4 T cells, T follicular helper cells, and regulatory T cells (Tregs) showed significantly reduced abundance in the higher-risk category (*P* < 0.05) (**Figure 7B**). Also, we selected gastric cancer tissues and their paired adjacent normal tissues to perform immunofluorescence staining for immune cells. The results showed that the positive expression levels of CD3^+^ T cells and CD3^+^CD4^+^ T helper cells in adjacent normal tissues were significantly higher than those in gastric cancer tissues (**Figure 8A, C, D**), suggesting that the number of total T cells and T helper cells in adjacent normal tissues was markedly greater than that in gastric cancer tissues. The proportion of CD68^+^CD86^+^ M1 macrophages in adjacent normal tissues was significantly higher than that in gastric cancer tissues (**Figures 8B, E**), while the proportion of CD68^+^CD206^+^ M2 macrophages was significantly lower (**Figures 8B, F**), indicating that adjacent normal tissues were enriched in M1-type macrophages with a markedly reduced proportion of M2 macrophages. Furthermore, the proportion of CD14^+^ monocytes in gastric cancer tissues was significantly higher than that in adjacent normal tissues (**Figures 8G, H**).

Furthermore, a correlation between prognostic genes and immune cells was observed; specifically, *AKAP12* exhibited a significant positive correlation with monocytes, with a correlation coefficient greater than 0.3 (*P* < 0.05) (**Figure 7C**). Immunofluorescent co-staining assays for *AKAP12* and CD14 (a surface marker of monocytes) showed that the number of *AKAP12*/CD14 double-positive stained cells was significantly higher in cancer tissues than in adjacent normal tissues. This experimental finding directly corroborated the existence of a correlation between *AKAP12* and monocytes at the tissue level (**Figures 8G, I**).

Subsequent analysis of immune checkpoint expression profiles in higher-risk and lower-risk GC categories revealed significant differences in the expression levels of 31 unique immune checkpoints (*P* < 0.05) (**Figure 7D**). These differences in immune checkpoint expression may indicate a relationship between disease severity and the modulation of immune responses.

Finally, TIDE scores were assessed for the higher-risk and lower-risk categories of patients with GC. The TIDE model predicts whether a tumor can resist current immunotherapies, such as checkpoint inhibitors, through immune evasion mechanisms. Consequently, a high TIDE score typically indicates a tumor with greater immune evasion capacity (36). TIDE scores were significantly higher in the higher-risk category (*P* < 0.001), suggesting that patients with a higher risk may have a poorer response to immunotherapy. Correlation analysis revealed a significant positive correlation between risk scores and TIDE scores (cor = 0.29, *P* < 0.0001) (**Figure 7E**), indicating that as the risk score increases, the tumor’s immune evasion capability is enhanced, potentially reducing the effectiveness of immunotherapy.

Further analysis of sensitivity to 138 commonly used chemotherapeutic and molecular targeting agents revealed significant IC50 differences between the higher-risk and lower-risk categories for 116 of these drugs (*P* < 0.05) (**Supplementary Table 2**). Imatinib and PLX4720 exhibited lower IC50 values in the higher-risk category (*P* < 0.0001), suggesting enhanced responsiveness to these agents in patients with higher risk profiles. In contrast, GW.441756, BAY.61.3606, and JNK.9L showed higher IC50 values in the higher-risk category (*P* < 0.0001), indicating a reduced therapeutic response to these drugs in patients at greater risk of disease progression (**Figure 7F**).

### 3.8 Expression validation of gastric cancer prognostic genes and functional validation of *CD36* and *KIT*

**Figure 9:**
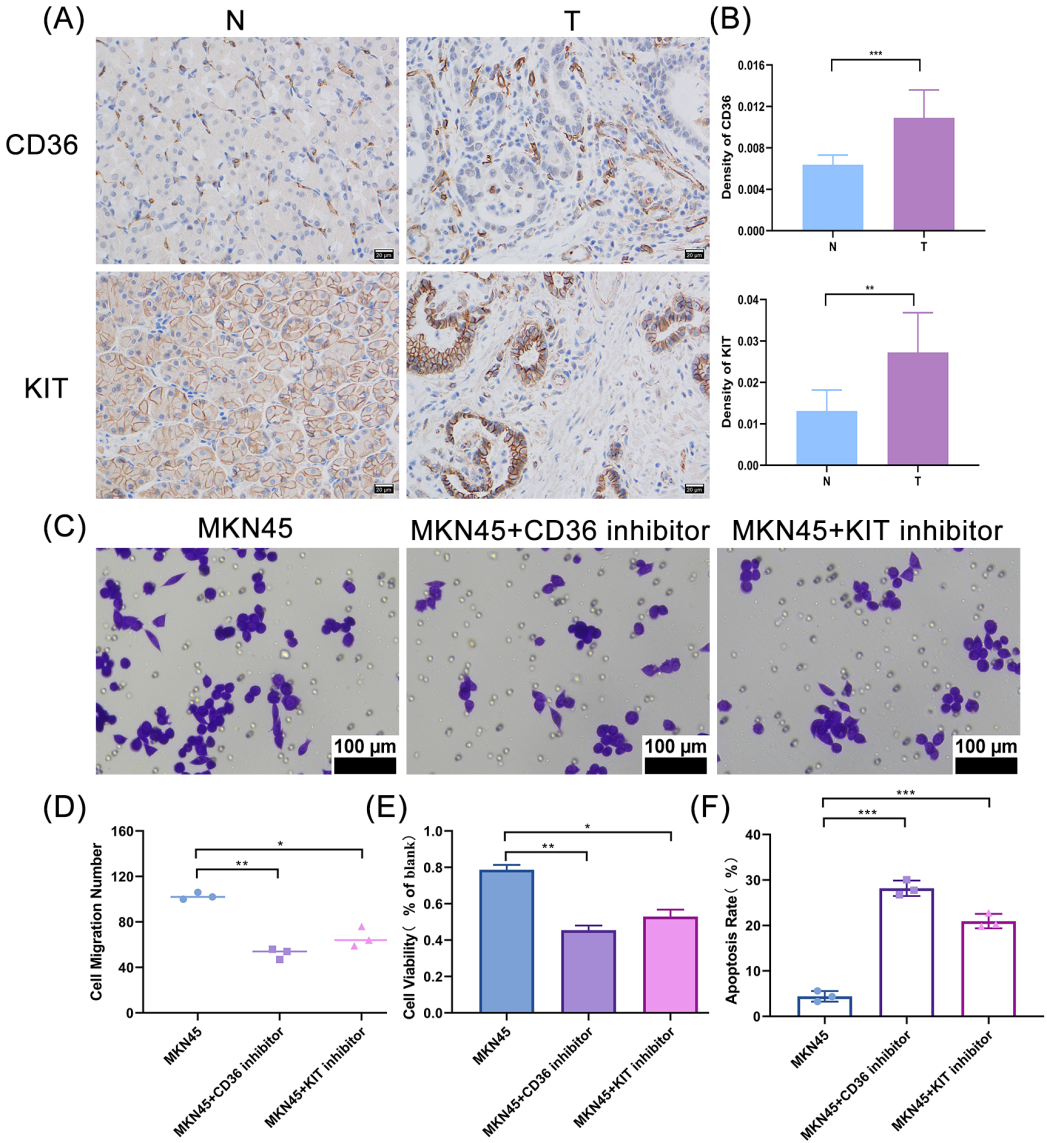
Detection of protein expression and functional verification of CD36 and KIT. **(A)** Immunohistochemical staining results of CD36 and KIT in gastric cancer tissues and adjacent non-tumor tissues. N: Adjacent normal, T: Tumor. Brown indicates positive expression of CD36 and KIT, blue indicates cell nuclei. **(B)** Quantitative analysis of immunohistochemical optical density of CD36 and KIT in gastric cancer tissues and adjacent non-tumor tissues. ** denotes *P* < 0.01, *** denotes *P* < 0.001. **(C)** Migration status of MKN45 cells after treated with CD36 and KIT inhibitors; **(D)** Number of migrated MKN45 cells after treated with CD36 and KIT inhibitors * denotes *P* < 0.05.** denotes *P* < 0.01. **(E)** Detection of cell proliferation ability in MKN45 cells treated with CD36/KIT inhibitors * denotes *P* < 0.05.** denotes *P* < 0.01 **(F)** Differential analysis of cell apoptosis level in MKN45 cells treated with CD36/KIT inhibitors.*** denotes *P* < 0.001.

Expression validation analysis demonstrated significant differences in the expression of prognostic genes (*PCOLCE2*, *CD36*, *ADAMTS8*, *KIT*, and *AKAP12*) between control and GC groups. The expression of *PCOLCE2*, *CD36*, *ADAMTS8*, and *KIT* was notably elevated in the control group compared to the GC group, while *AKAP12* exhibited higher expression in the GC group (**Supplementary Figure 7A**). All five prognostic genes showed statistically significant differences between the two groups (*P* < 0.05).We further selected *CD36* and *KIT* for protein-level validation via immunohistochemical (IHC) assays. The results showed that the protein expression levels of both CD36 and KIT in gastric cancer tissues were significantly higher than those in the control group (**Figures 9A, B**), which was contrary to the findings of qPCR assays. Based on the above protein-level detection results, we performed subsequent functional validation using *CD36* and *KIT* inhibitors in gastric cancer MKN45 cells, confirming that the two inhibitors could downregulate the protein expression of *CD36* and *KIT* in MKN45 cells, respectively (**Supplementary Figure 8**). Furthermore, the downregulation of *CD36/KIT* protein expression significantly inhibited the migration (**Figures 9C, D**) and proliferation (**Figure 9E**) of MKN45 cells, and promoted their apoptosis (**Figure 9F**). Collectively, these results suggest that the prognosis-related genes *CD36* and *KIT* can promote the initiation and progression of gastric cancer when highly expressed in gastric cancer cells.

### 3.9 Functional validation of *CD36 and KIT* genes in regulating the TME and PA pathway in gastric cancer

**Figure 10:**
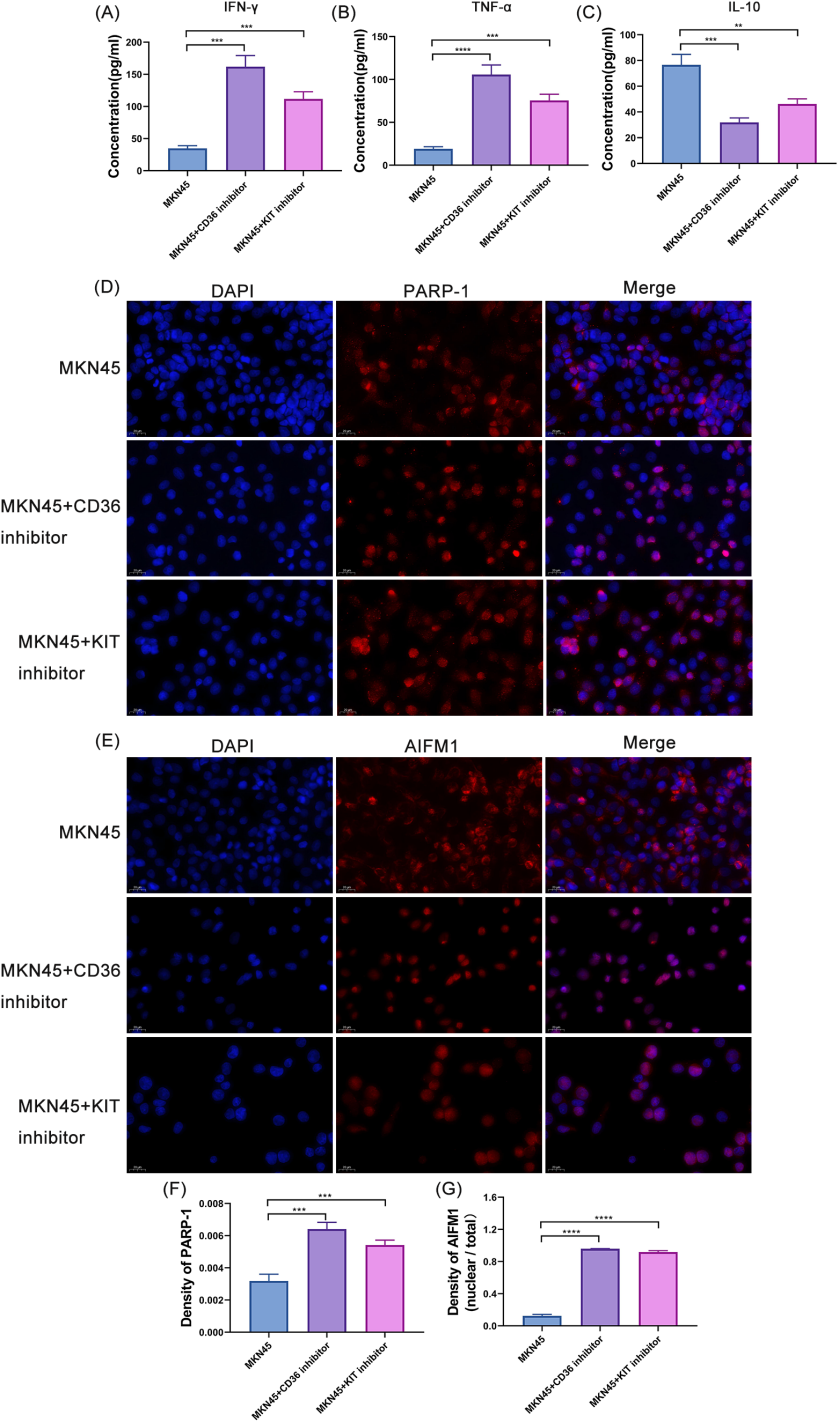
Correlation analysis of *CD36, KIT* with TME and PA. **(A)** Detection of IFN-γ secretion level in MKN45 cells after treatment with CD36 and KIT inhibitors. *** denotes *P* < 0.001. **(B)** Detection of TNF-α secretion level in MKN45 cells after treatment with CD36 and KIT inhibitors. *** denotes *P* < 0.001.**** denotes *P* < 0.0001. **(C)** Detection of IL-10 secretion level in MKN45 cells after treatment with CD36 and KIT inhibitors.** denotes *P* < 0.01. *** denotes *P* < 0.001. **(D)** Immunofluorescence staining results of PARP-1 in MKN45 cells after treatment with CD36 and KIT inhibitors. **(E)** Immunofluorescence staining results of AIFM1 in MKN45 cells after treatment with CD36 and KIT inhibitors. **(F)** Differential analysis of immunofluorescence optical density of PARP-1 in MKN45 cells after treatment with CD36 and KIT inhibitors. *** denotes *P* < 0.001. **(G)** Differential analysis of the ratio of nuclear to total immunofluorescence optical density of AIFM1 in MKN45 cells after treatment with CD36 and KIT inhibitors.**** denotes *P* < 0.0001.

To clarify the correlations between *CD36*, *KIT* and TME as well as the PA pathway, we performed ELISA-based cytokine detection and immunofluorescence staining for PARP-1 and AIFM1 in MKN45 cells following treatment with *CD36* or *KIT* inhibitors. ELISA results showed that the secretion levels of TNF-α and IFN-γ in the *CD36* and *KIT* inhibitor groups were significantly higher than those in the control group, while the secretion level of IL-10 was significantly lower (**Figures 10A–C**). Immunofluorescence staining results revealed that PARP-1 was predominantly localized in the cell nucleus with a uniform diffuse distribution in gastric cancer MKN45 cells of the control group. After separate treatment with *CD36* or *KIT* inhibitors, the nuclear fluorescence intensity of PARP-1 was significantly increased, and its distribution pattern was converted to a typical punctate aggregation associated with DNA damage foci (**Figures 10D, F**). Meanwhile, the ratio of nuclear to total fluorescence intensity of AIFM1 was markedly elevated (**Figures 10E, G**).

## 4 Discussion

GC represents a major global health burden, with a high incidence in regions such as Asia, Eastern Europe, and Central America, where current treatments often yield unsatisfactory outcomes (37, 38). The TME and PA are crucial in GC progression and response to treatment (39, 40). This study identified seven TME- and PA-related prognostic genes (*EGF*, *PCOLCE2*, *CD36*, *ADAMTS8*, *CIDEC*, *KIT*, and *AKAP12*) using bioinformatics and machine learning techniques, constructing a prognostic model with good predictive performance, offering new insights to refine GC treatment strategies.

A-kinase anchoring protein 12 (*AKAP12*) is a recognized tumor suppressor that regulates the cell cycle and cytoskeletal structure by binding to various protein kinases. Through its ability to prevent PKC activation, *AKAP12* inhibits angiogenesis, cell proliferation, and cancer cell invasion, playing a key role in cancer suppression. Beyond its role as a tumor suppressor, *AKAP12* is closely associated with the progression and prognosis of gastric cancer by regulating the tumor immune microenvironment—including immune cell infiltration, immune pathways, and immunomodulatory factors (41). In addition, the function of *AKAP1*2 is also regulated by non-coding RNAs, such as the LINC00163/miR-183 axis, a regulatory mechanism that plays an important role in the invasion and metastasis of gastric cancer (42). In GC, *AKAP12* expression is significantly reduced, and this low expression correlates with the infiltration of CD4+ T cells and macrophages. Furthermore, decreased *AKAP12* levels are linked to reduced cancer cell invasion and metastasis, highlighting its potential as a therapeutic target (41, 43).

Procollagen C-Endopeptidase Enhancer 2 (*PCOLCE2*) is an ECM glycoprotein that enhances the activity of procollagen C-terminal proteases, a process essential for ECM remodeling. Bioinformatics analyses have identified *PCOLCE2* as a key gene influencing clinical prognosis in various cancers, including GC and bladder cancer (44–46).Its expression is closely associated with changes in the tumor immune microenvironment, and particularly its elevated expression in cancer-associated fibroblasts (CAFs) makes it a potential therapeutic target in gastric cancer research (45).

Platelet glycoprotein 4 (*CD36*), a scavenger receptor primarily involved in lipid metabolism, also plays pleiotropic roles in angiogenesis, inflammation, and metabolic disorders such as diabetes and obesity (47).While there is currently no literature reporting a direct interaction between *CD36* and PA, CD36 may function as a key receptor during cell death processes, regulating signaling pathways related to immune responses and cell death (48). This regulatory role might indirectly influence PA as a form of cell death. In GC, *CD36* functions as a fatty acid receptor and co-receptor for Toll-like receptors, promoting epithelial-mesenchymal transition (EMT) and metastasis. Notably, the *CD36*-BATF2/MYB signature has been shown to predict the response of patients with GC to anti-PD-1 immunotherapy, underscoring its potential as a biomarker for immunotherapeutic efficacy (49). Furthermore, *CD36* influences the prognosis of gastric cancer by modulating lipid metabolism and immune cell infiltration within the tumor microenvironment, playing a significant role in immune evasion and tumor immunoevasion mechanisms (50). In summary, *CD36* plays a crucial role in the immune escape and therapeutic response of gastric cancer, holding promise as a potential therapeutic target.

A Disintegrin and Metalloproteinase with Thrombospondin Motifs (*ADAMTS*) family plays dual roles in various cancers, including GC, functioning both as tumor suppressors and oncogenes (51). Specifically, *ADAMTS8* is highly expressed in GC tissues, and its upregulation is strongly associated with poor prognosis, suggesting its potential as a prognostic biomarker (52). Studies have indicated that the methylation of *ADAMTS8* is closely associated with lymph node metastasis in gastric cancer, suggesting its important role in the development and progression of gastric cancer (53). Additionally, the Immunoreactivity Score (IRS) of *ADAMTS8* is significantly elevated in both cancerous gastric tissues and metastatic lymph nodes, further demonstrating its value in gastric cancer occurrence and lymphatic metastasis (54). In conclusion, *ADAMTS8* holds significant importance as a potential prognostic biomarker.

The epidermal growth factor (*EGF*) gene plays a crucial role in the regulation of cell growth by *EGF* receptor. Particularly in tumorigenesis, overexpression of the *EGF* receptor may promote tumor progression (55). In gastric cancer, the *EGF* signaling pathway, especially under the regulation of DENND1A and Rab35, influences the migration, invasion, and prognosis of gastric cancer cells (56). Furthermore, dysregulation of *EGF* signaling is one of the potential mechanisms underlying gastric cancer development. During gastric mucosal lesions, the expression and function of *EGF* may alter cell differentiation and gastric gland architecture, thereby creating conditions conducive to cancer initiation (57). Therefore, the *EGF* signaling pathway represents a promising therapeutic target in gastric cancer treatment.

Cell death-inducing DFF45-like effector C (*CIDEC*) is a protein that plays a significant role in diet-induced adipose tissue inflammation, and its expression is closely linked to arterial inflammation and vascular remodeling in type 2 diabetes (58). Moreover, *CIDEC* is important for lipid accumulation and lipid droplet protection, particularly in hepatic lipid metabolism (59). Recent studies further indicate that in gastric cancer, *CIDEC* may influence patient survival outcomes by regulating the infiltration of CD8^+^T cells within the tumor immune microenvironment, suggesting its potential as a biomarker for immunotherapy and prognostic evaluation in gastric cancer (60).

The receptor tyrosine kinase *KIT* (also known as c-*KIT* or CD117) is a key regulator of cell proliferation and viability. Mutations or overexpression of *KIT* are linked to several diseases, including gastrointestinal stromal tumors (GISTs), highlighting its significance in cancer biology (61).

In this study, patients were stratified into high-risk (n = 175) and low-risk (n = 175) groups based on the median prognostic gene risk score. Model validation revealed that risk scores were positively correlated with patient mortality and negatively correlated with survival rates in both the training and validation cohorts. Specifically, the high-risk group exhibited significantly higher mortality and lower survival rates compared to the low-risk group, confirming the strong association between the prognostic genes and patient survival outcomes. Compared to the models developed by Khan and Zhai, our model demonstrated superior performance, with AUC values exceeding 0.6 in both the training and validation sets, surpassing their respective AUC values. These results further validate the effectiveness and accuracy of our prognostic gene model (62, 63). To assess the correlation between risk scores and clinical parameters, the distribution of patient characteristics (including age, gender, and TNM stage) across high- and low-risk groups was examined. Significant differences were observed in T1/T2 staging and in patients aged above or below 60 years (*P* < 0.05), indicating that the gene-based risk score effectively reflects tumor invasiveness and age-related factors. To further elucidate the risk determinants in GC and verify the prognostic gene signature, univariate and multivariate Cox regression analyses were performed, incorporating patient risk scores and clinical parameters. The analysis revealed that, in addition to age, N stage, and M stage, the risk score served as an independent prognostic indicator. A nomogram integrating these four independent predictors was subsequently developed, demonstrating strong concordance between predicted and observed survival outcomes (AUC > 0.7), confirming its robust predictive accuracy. These findings highlight the nomogram’s clinical utility in personalizing treatment strategies and therapeutic decision-making. The developed prognostic model holds promising potential for clinical application in GC management.

To compare immune cell infiltration patterns between high- and low-risk groups, the CIBERSORT algorithm was applied to quantify 22 immune cell subtypes in GC samples from the training cohort. The analysis revealed increased infiltration of memory B cells, naïve B cells, M2 macrophages, resting mast cells, and monocytes in high-risk patients. Notably, M2-polarized macrophages—activated by IL-13, IL-4, TGF-β, and IL-10, are known to promote tumor progression (64). High infiltration of M2 macrophages in high-risk patients is closely associated with immune evasion and tumor progression in gastric cancer, which is consistent with the conclusions of existing studies—Cisplatin-induced activation of hypoxia-inducible factor 1α (HIF1α) signaling can drive M2 macrophage polarization by regulating the STAT3 pathway, thereby enhancing tumor immune tolerance and promoting chemoresistance. These findings suggest that the HIF1α/STAT3 pathway involved in M2 polarization is a potential therapeutic target for gastric cancer (64). In addition, monocytes infiltration was increased in the high-risk group. Monocytes exhibit strong plasticity and can differentiate into protumor M2 macrophages or antitumor M1 macrophages under the regulation of tumor microenvironment (TME) signals (65). Furthermore, this study found a strong positive correlation between AKAP12 and monocyte abundance, suggesting that AKAP12 may be involved in the remodeling of the gastric cancer immune microenvironment by regulating monocyte differentiation (65), which provides new clues for the formation mechanism of the immunosuppressive microenvironment in the high-risk group.

In contrast, patients in the low-risk category exhibited higher levels of M0 macrophages, stimulated mast cells, resting NK cells, activated memory CD4 T cells, follicular helper T cells, and Tregs. NK cells play a pivotal role in directly killing tumor cells, even those resistant to CD8^+^ T cell recognition (64). Existing studies have demonstrated that death-associated protein kinase 1 (DAPK1) can enhance the cytotoxic activity of NK cells by inhibiting the IKKβ/CSN5/PD-L1 axis, thereby suppressing immune evasion in gastric cancer (64). The high infiltration of NK cells in the low-risk group may therefore represent a key reason for the weaker immune evasion capacity of gastric cancer in this subgroup. Activated CD4 T cells are particularly valuable for immunotherapy, as many therapeutic strategies rely on their activation and subsequent immune modulation (64). Tfh cells enhance B cell functionality within the TME, thereby amplifying cytotoxic CD8^+^ T cell responses. Clinical observations consistently associate elevated Tfh populations with improved patient outcomes. These findings also provide an immunological explanation for the more favorable prognosis of patients in the low-risk group.

The results of tissue-level immunofluorescence staining were highly consistent with the aforementioned infiltration characteristics: the positive expression levels of CD3^+^T cells and CD3^+^CD4^+^ helper T cells, as well as the proportion of CD68^+^CD86^+^ M1-type macrophages, were significantly lower in gastric cancer tissues than in adjacent normal tissues, while the proportions of CD68^+^CD206^+^ M2-type macrophages and CD14^+^ monocytes were significantly higher. These findings were consistent with the features of the high-risk group—high infiltration of M2-type macrophages and monocytes, and low infiltration of activated CD4^+^T cells—and also aligned with the conclusions of existing studies in the field of gastric cancer tumor immune microenvironment (64). Additionally, this study performed double immunofluorescence staining of *AKAP12* and CD14 in gastric cancer tissues and their paired paracancerous tissues, and found that the number of *AKAP12*/CD14 double-positive cells was significantly higher in gastric cancer tissues. This result directly confirmed the correlation between *AKAP12* and monocytes at the tissue level, providing experimental evidence for subsequent investigations into their regulatory mechanisms.

Abnormal expression of immune checkpoint molecules in the TME constitutes another crucial mechanism underlying immune evasion in gastric cancer. Existing studies have confirmed that tumor cells with high PD-L1 expression can enhance immunosuppressive activity by attenuating the cytotoxicity of T cells, monocytes, NK cells and macrophages, enabling tumors to escape immune surveillance and elimination, and leading to resistance to anti-PD-1/PD-L1 therapy (66). To further verify the immune evasion potential of the high- and low-risk groups, the TIDE algorithm, which exhibits excellent specificity and sensitivity, was adopted in this study for assessment. Comparative analysis revealed significantly elevated TIDE scores in high-risk patients compared to low-risk counterparts, suggesting enhanced immune escape mechanisms and immune dysfunction in high-risk subgroup (67).These findings highlight the heterogeneous nature of the immune microenvironment across different risk categories, further validating the accuracy of the prognostic signature in this study and providing a novel perspective for elucidating the molecular mechanisms underlying immune evasion in gastric cancer.

Additionally, a drug sensitivity analysis was conducted based on the prognostic gene model. The results indicated that the high-risk group exhibited greater sensitivity to Imatinib and PLX4720 compared to the low-risk group. Imatinib (Gleevec) is a groundbreaking anti-cancer drug primarily used to treat chronic myelogenous leukemia (CML) and GISTs. It works by inhibiting specific tyrosine kinases, such as BCR-ABL and PDGF receptors, thereby blocking tumor cell growth signals (68–70). In another study, Imatinib was identified to hold therapeutic potential for gastric cancer, particularly by inducing apoptosis via the generation of reactive oxygen species (ROS) and activation of c-Jun N-terminal kinase (JNK). PLX4720 is a kinase inhibitor targeting the BRAF V600E mutation, commonly found in cancers such as melanoma and thyroid cancer. PLX4720 exerts its anti-cancer effects by selectively inhibiting BRAF kinase activity (71–73).In the treatment of thyroid cancer, PLX4720 can effectively inhibit the proliferation, migration and invasion of tumor cells driven by the BRAF(V600E) mutation, and exhibits remarkable therapeutic potential especially for undifferentiated thyroid cancer (ATC) (73). However, the development of resistance to cancer therapeutics constitutes a key challenge in clinical treatment. PLX4720 is closely associated with the development of cancer resistance (74), and further investigation into its resistance mechanisms is of great significance for improving therapeutic efficacy. Thus, Imatinib and PLX4720 could be considered potential treatment options for GC in the high-risk group, with BCR-ABL, PDGF receptors, and BRAF V600E as potential therapeutic targets for GC, further investigation into the resistance mechanisms of these targets will help optimize existing therapeutic strategies.

RT-qPCR was used to verify the expression differences of five prognostic genes (*PCOLCE2, CD36, ADAMTS8, KIT and AKAP12*) between gastric cancer and adjacent normal tissues, and statistically significant differences in the expression levels of all five genes were observed between the two groups, which preliminarily confirmed their association with gastric cancer oncogenesis. Conversely, *AKAP12* was highly expressed in gastric cancer tissues, which was inconsistent with both the gene expression data (**Supplementary Figure 7B**) and previous studies. This discrepancy might be attributed to limited sample size and heterogeneity in sample sources. We then selected *CD36* and *KIT*, two well-recognized functional genes in oncology—*CD36* is involved in tumor lipid metabolism, invasion and metastasis (48),and *KIT* is a tyrosine kinase receptor that regulates tumor cell proliferation, with its abnormal protein expression closely correlated with clinical prognosis (61)—and verified their protein expression levels by immunohistochemistry (IHC). The IHC results were inconsistent with those of RT-qPCR, a phenomenon speculated to be associated with differences in post-transcriptional regulation and translational modification. Specifically, specific microRNA regulation, epigenetic modification or mRNA degradation mechanisms may exist in gastric cancer tissues, leading to the uncoupling of transcriptional and protein expression levels of *CD36* and *KIT (*75). As the direct executors of biological activities, proteins can more truly reflect the biological functions of genes by their expression levels (75), this also provides the basis for conducting inhibitor intervention experiments based on protein-level results in subsequent studies.

*CD36/KIT* inhibitors could specifically downregulate the expression of the corresponding proteins in gastric cancer MKN45 cells, significantly inhibit cell proliferation and migration, and promote apoptosis. These findings confirmed that both *CD36* and *KIT* are oncogenes in gastric cancer, and targeted blockade of their expression can suppress the malignant phenotype of gastric cancer cells, providing cell-level experimental evidence for their potential as targeted therapeutic. Further studies revealed that inhibitor treatment led to increased secretion of the pro-inflammatory cytokines TNF-α and IFN-γ and decreased secretion of the anti-inflammatory cytokine IL-10 in cells, suggesting that blocking the *CD36*/*KIT* pathway can remodel the cytokine secretion profile of the gastric cancer tumor microenvironment and shift it from an immunosuppressive to an immune-activated state. Notably, this study is the first to identify a close association between the *CD36*/*KIT* pathway and the PA programmed cell death pathway in gastric cancer. Immunofluorescence results showed that the inhibitors could activate PARP-1 and induce AIFM1 nuclear translocation, indicating that they may trigger PA in gastric cancer cells by inducing DNA damage to activate PARP-1 and subsequent AIFM1 nuclear translocation. This finding uncovers a novel molecular mechanism underlying gastric cancer cell death induced by *CD36/KIT* inhibitors and also provides a new strategy for the combined application of these inhibitors with chemotherapeutic drugs to enhance the cytotoxic effect on gastric cancer cells.

In this study, we integrated bioinformatics analysis with the predictive RSF+plsRcox model and experimental validation to screen gastric cancer prognosis-related genes associated with the TME and PA pathway, namely *EGF, PCOLCE2, CD36, ADAMTS8, CIDEC, KIT and AKAP12*, and further constructed a prognostic evaluation model. Meanwhile, we clarified the oncogenic roles of *CD36* and *KIT* in gastric cancer, as well as their dual molecular mechanisms in regulating the TME and PA pathway, which provides novel experimental evidence for prognostic assessment and precise targeted therapy of gastric cancer. However, this study has several limitations. First, the predictive model established based on bioinformatics approaches remains at the theoretical stage, which requires validation in an expanded clinical cohort and further evaluation of its clinical applicability for diagnosis and prognosis of gastric cancer. Second, the retrospective study design may introduce selection bias; prospective studies should be conducted in the future to verify the model accuracy and address potential biases. Third, the prediction of gastric cancer response to immunotherapy in this study relied solely on TIDE scores without incorporating actual clinical data from patients receiving immunotherapy, which may lead to certain biases in the prediction results. Subsequent studies need to include real-world data of immunotherapy-treated patients to validate the clinical predictive value of TIDE scores for the efficacy of gastric cancer immunotherapy. Fourth, the differential effects of immune cell infiltration and immune checkpoint molecule expression detected in this study were modest, and the biological significance of some indicators remains unclear. For instance, the correlation coefficient between *AKAP12* and monocytes was only 0.3, and its specific regulatory mechanisms and biological value require further in-depth experimental investigation. Fifth, the small sample size for RT-qPCR assays may introduce random errors, and the results may not fully represent the overall expression characteristics of gastric cancer patients.

## 5 Conclusions

This study innovatively integrated TME-RGs with PA-RGs to develop a machine learning-driven prognostic model for GC. Seven key genes (*EGF*, *PCOLCE2*, *CD36*, *ADAMTS8*, *CIDEC*, *KIT*, and *AKAP12*) were identified as significant prognostic markers, with the RSF + plsRcox model showing strong predictive performance (C-index > 0.6). A clinical nomogram incorporating risk scores, age, and TNM stage demonstrated high accuracy (AUC > 0.7) in survival prediction. High-risk patients exhibited an immunosuppressive TME with increased M2 macrophages, immune checkpoint expression, and higher TIDE scores, suggesting a poorer response to immunotherapy. Tissue-level assays further demonstrated the consistency of GC tissue immune infiltration features with the high-risk group, and confirmed the correlation between *AKAP12* and monocytes. Drug sensitivity analysis indicated the potential efficacy of Imatinib and PLX4720 in high-risk cases. RT-qPCR validated differential gene expression in GC tissues. And subsequent experimental validations confirmed that high expression of *CD36/KIT* promotes GC cell proliferation and migration while inhibiting apoptosis; their inhibition remodels the TME cytokine profile, and this study is the first to discover that *CD36/KIT* inhibition activates the PA pathway to induce GC cell death. Collectively, this study establishes a novel risk stratification framework for GC, provides potential prognostic markers and therapeutic targets, and offers experimental evidence for personalized treatment of GC patients.

## Data Availability

The datasets presented in this study can be found in online repositories. The names of the repository/repositories and accession number(s) can be found in the article/**Supplementary Material**.
