# Supplementary material for: Prognostic integration of tumor microenvironment and parthanatos-related genes in gastric cancer: a machine learning-driven risk model and immune landscape profiling
Source: Front Immunol. 2026 Feb 20;17:1636331. doi: 10.3389/fimmu.2026.1636331 (PMC12962909; doi:10.3389/fimmu.2026.1636331)
Supplement: Supplementary file 1 [file DataSheet1.pdf]

frontiers

Supplementary Material

1 Supplementary Figure

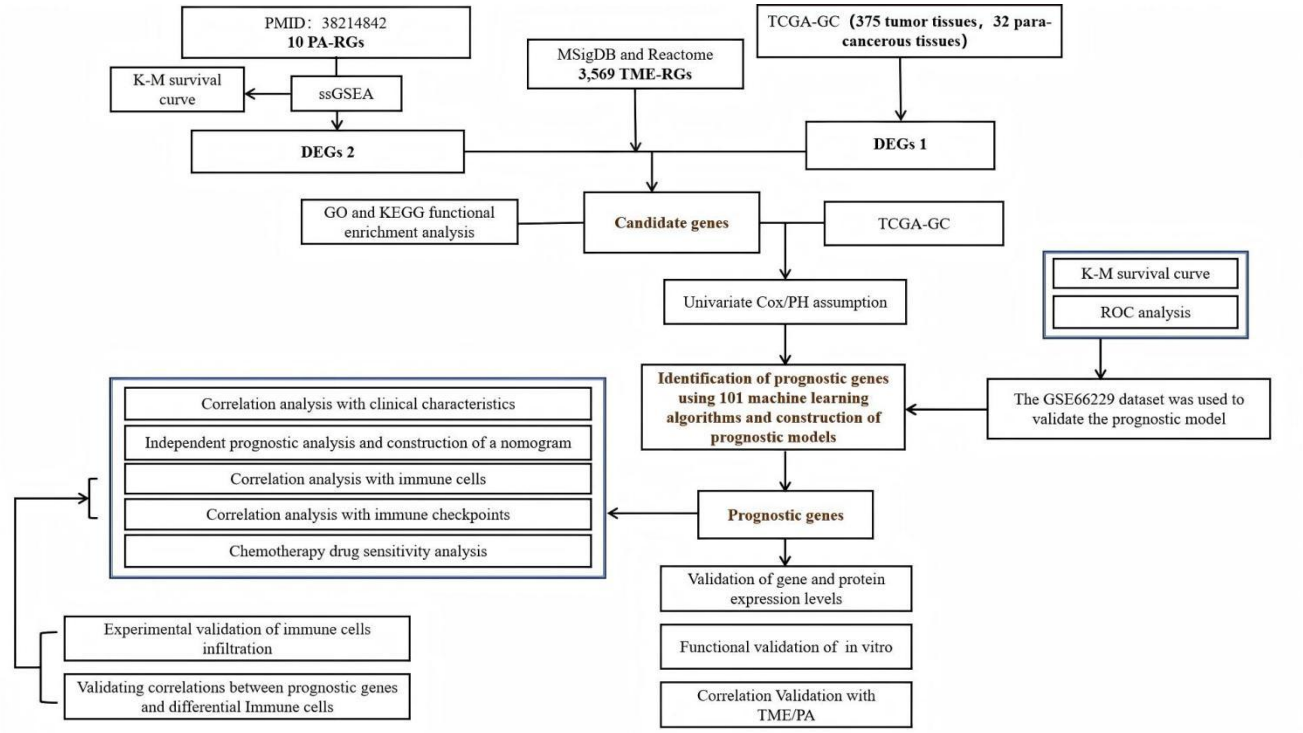

Supplementary Figure 1 Flowchart of the research design.

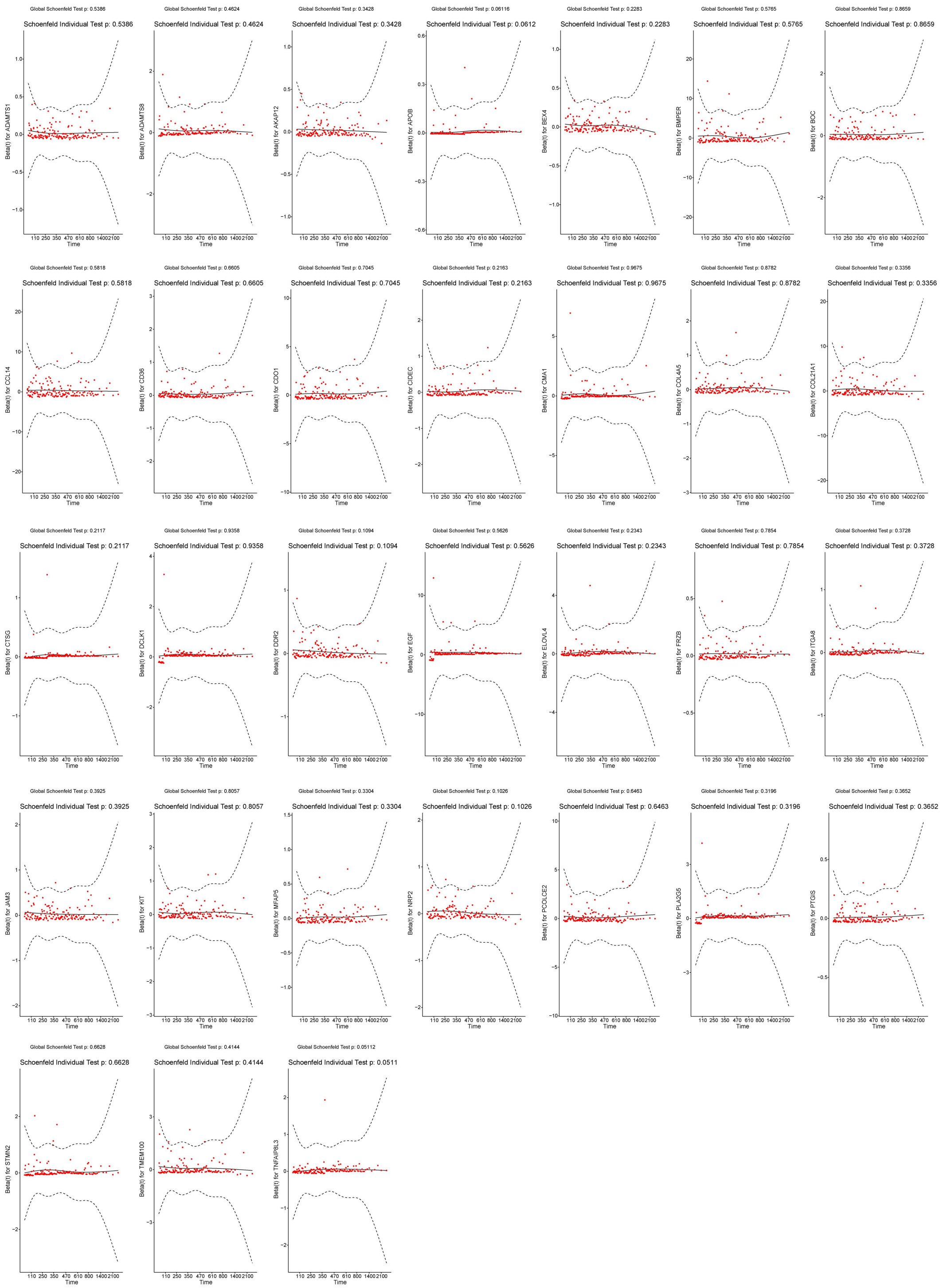

Supplementary Figure 2 Analysis of differentially expressed genes and prognostic significance of PA-RGs scoring in gastric cancer.

(A) (B)

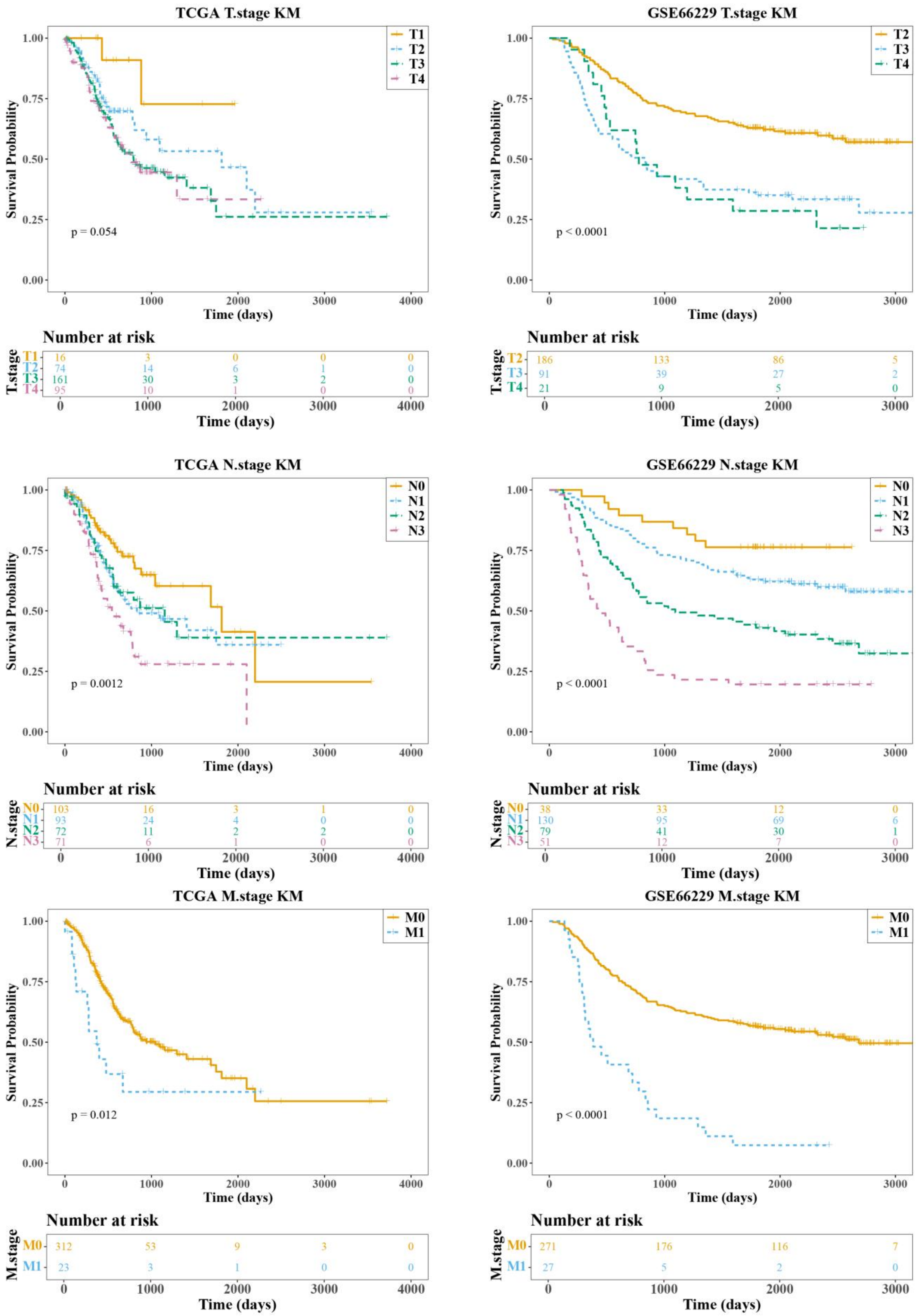

Supplementary Figure 3 KM analysis of TNM staging. **(A)** Training set. **(B)** Validation set.

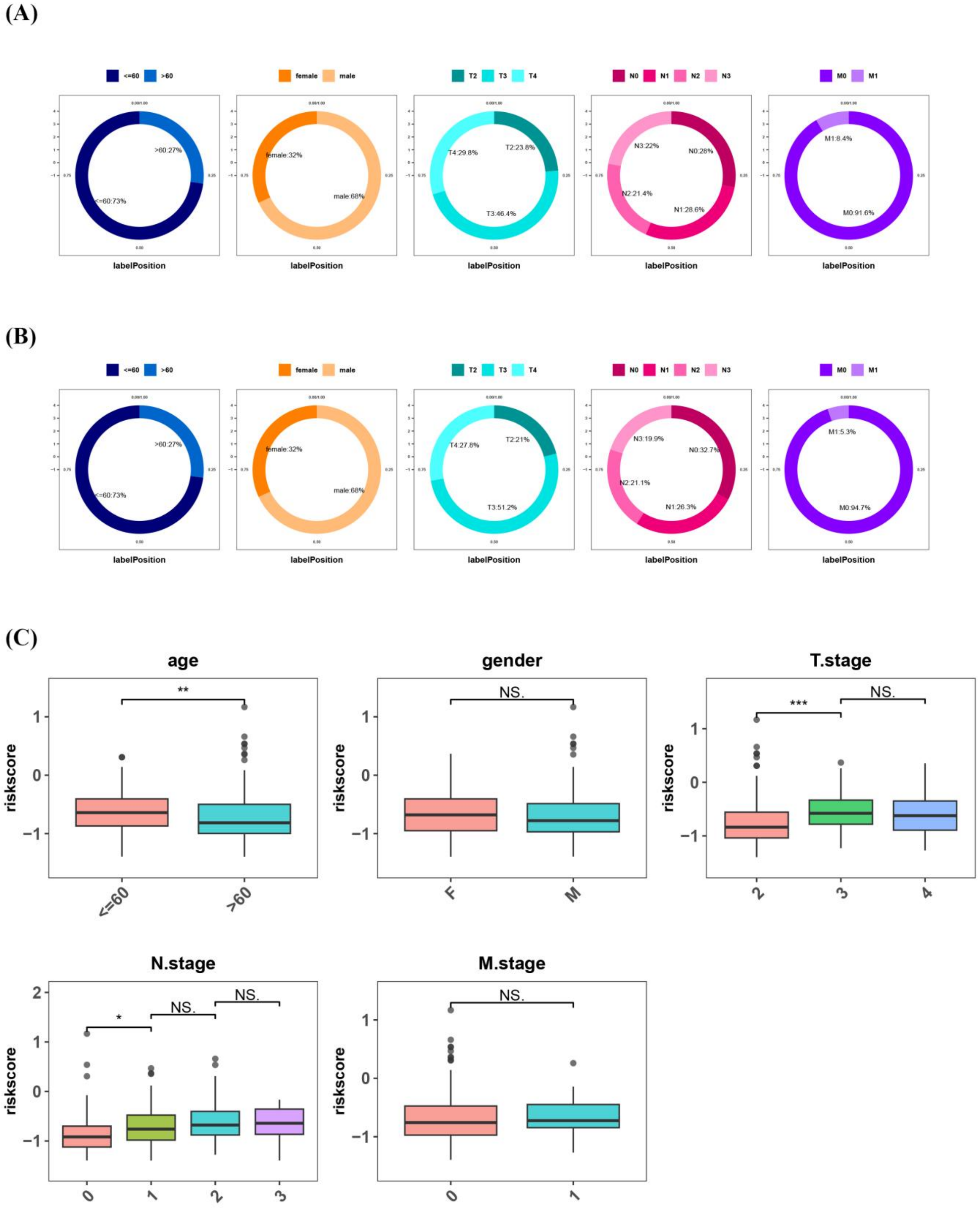

Supplementary Figure 4 Association analysis of risk score with clinical characteristics in the validation set. (A-B) Distribution of patients in high- and low-risk groups across different clinical subgroups. Panel A shows the distribution for the high-risk group, and Panel B for the low-risk group. (C) Differences in risk scores among different clinical subgroups.

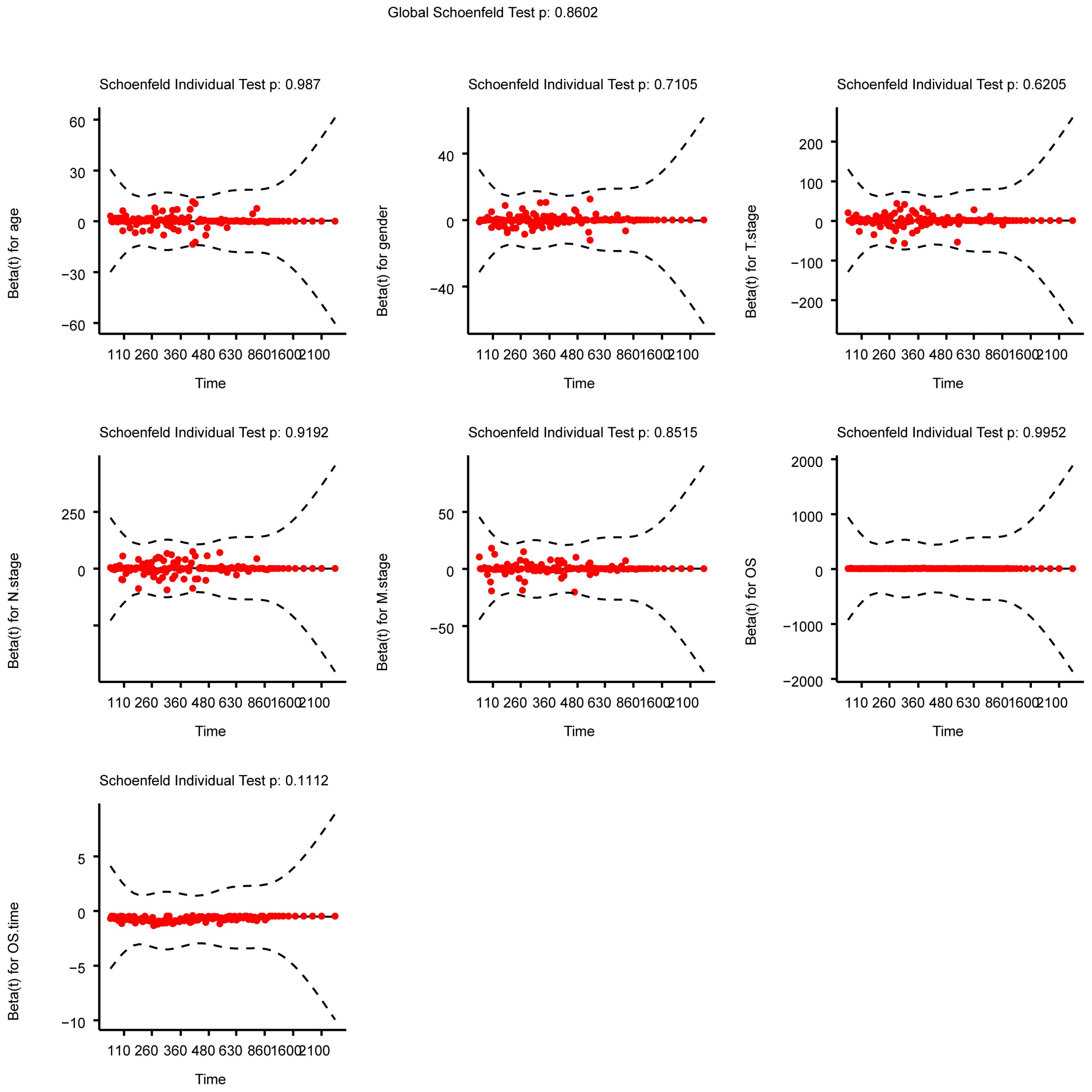

Supplementary Figure 5 Selection and functional enrichment of candidate genes.

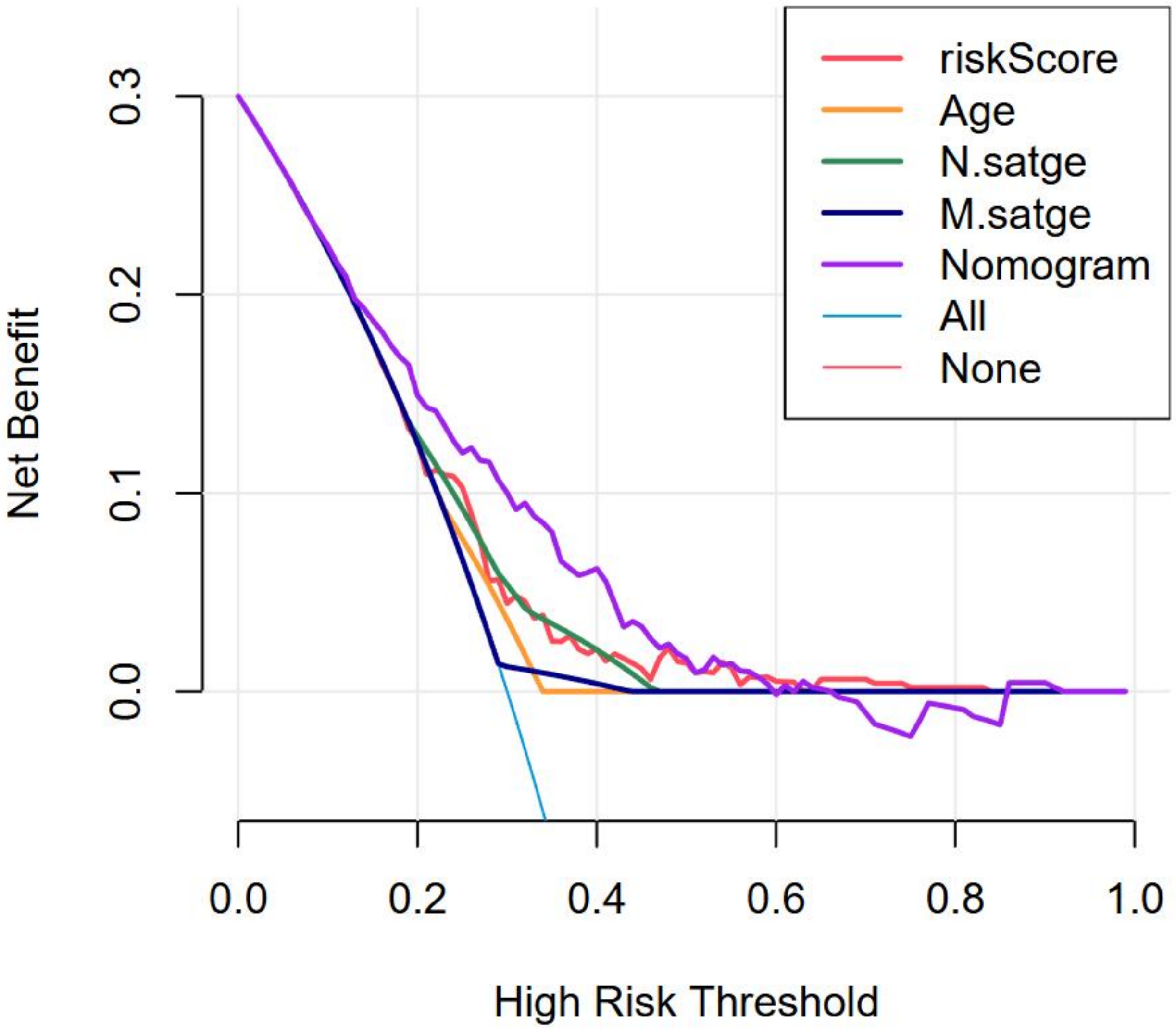

Supplementary Figure 6 Decision curve analysis (DCA) for the nomogram, gene risk score and various clinical indicators.

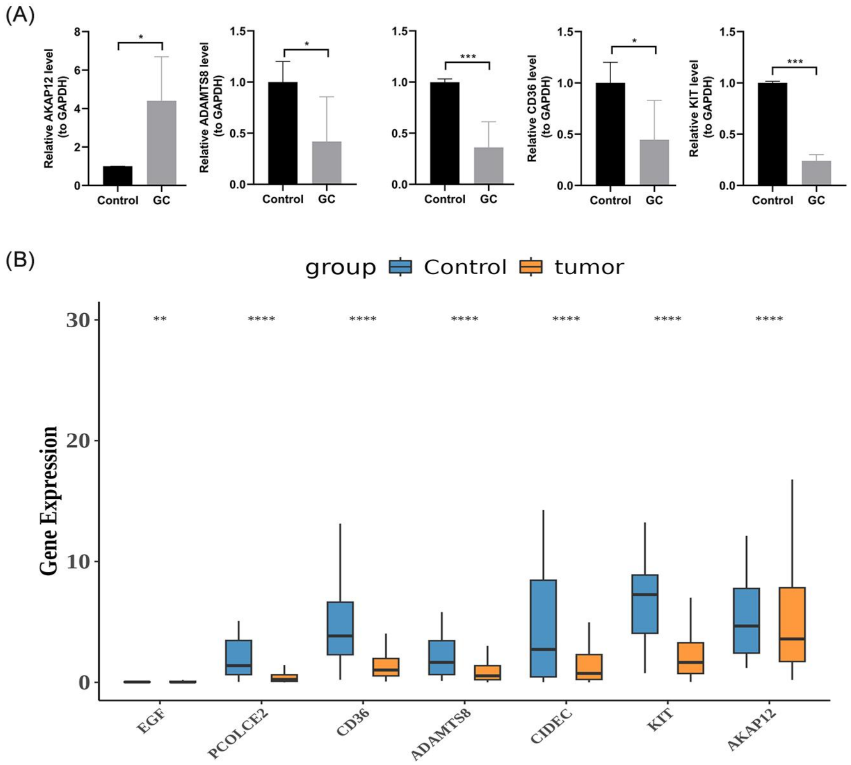

Supplementary Figure 7 Bar plot of differential expression of key genes between gastric cancer and control groups. **(A)** Expression validation analysis showing differences in prognostic genes (*KIT, CD36, AKAP12, ADAMTS8, and PCOLCE2*) between adjacent GC tissue samples and GC tissue samples (RT-qPCR). **(B)** Differential expression of prognostic genes (*EGF, POLCE2, CD36, ADAMTS8, CIDEC, KIT, AKAP12*) between gastric cancer and control groups (RNA-seq).

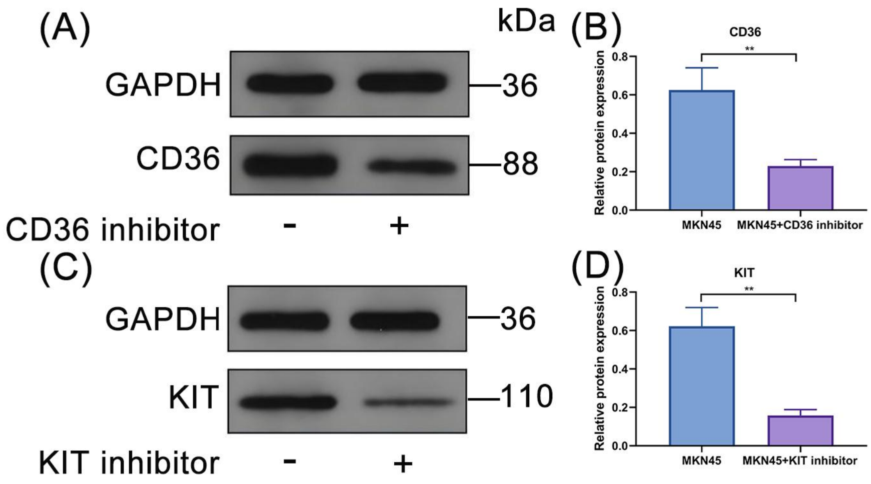

Supplementary Figure 8 Western blot analysis of related proteins in gastric cancer MKN45 cells treated with *CD36* and KIT inhibitors. **(A)** Protein bands of GAPDH and CD36. **(B)** Relative protein expression of CD36 (gray value normalization). **(C)** Protein bands of GAPDH and KIT. **(D)** Relative protein expression of KIT (gray value normalization)

2 Supplementary Tables

Supplementary Table 1 List of PCR primers

Primer	Sequences 5'-3'
PCOLCE2 F	TTCTCCGCTGCTGAACCAAA
PCOLCE2 R	TAGCATCGTTGACTTCCCCG
CD36 F	GGGTGTGGAAGGTTGTGTTG
CD36 R	TAAGCAGGTCTCCAACTGGC
ADAMTS8 F	AGGTGATTGATGGCACCCTG
ADAMTS8 R	AGCCATAATTGGTGGGGGTG
KIT F	GGAAGCCTCTTCCCAAGGAC
KIT R	GCTGGCCTCACTTTCAGGAT
AKAP12 F	AAGCAAATGGGGACTCGGAC
AKAP12 R	AGCCTGCGAATGAAGGAGAC
GAPDH F	CGAAGGTGGAGTCAACGGATTT
GAPDH R	ATGGGTGGAATCATATTGGAAC

Supplementary Table 2 Supplementary Table 2 GO analysis of DEGs

	high_group_res	low_group_res	padj	pvalue	log2FoldChange	drugs	sig	direction
GW.441756	4.286	4.285	4.64E-30	3.37E-32	0.002111014	GW.441756	GW.441756****	Sensitive to low-risk group
BAY.61.3606	2.45	2.293	2.20E-29	4.77E-31	0.180362432	BAY.61.3606	BAY.61.3606****	Sensitive to low-risk group
PLX4720	4.878	4.975	2.20E-29	4.43E-31	-0.12782	PLX4720	PLX4720****	Sensitive to high-risk

					2587			group
JNK.9L	-0.2405	-0.2861	2.83E-29	8.20E-31	0.057240559	JNK.9L	JNK.9L****	Sensitive to low-risk group
Imatinib	4.962	5.159	3.41E-28	1.24E-29	-0.304630906	Imatinib	Imatinib****	Sensitive to high-risk group
Roscovitine	4.862	4.725	6.05E-28	2.63E-29	0.178676344	Roscovitine	Roscovitine****	Sensitive to low-risk group
GNF.2	4.577	4.699	8.46E-28	4.29E-29	-0.1769815	GNF.2	GNF.2****	Sensitive to high-risk group
AZD8055	0.3468	0.3469	1.15E-27	6.67E-29	-0.000128218	AZD8055	AZD8055****	Sensitive to high-risk group
A.443654	-0.567	-0.6896	1.99E-27	1.29E-28	0.147961382	A.443654	A.443654****	Sensitive to low-risk group
Vinorelbine	-3.878	-3.925	4.80E-27	3.48E-28	0.057763234	Vinorelbine	Vinorelbine****	Sensitive to low-risk group
Bosutinib	2.767	2.72	6.03E-27	5.06E-28	0.05858378	Bosutinib	Bosutinib****	Sensitive to low-risk group
AMG.706	4.211	4.336	6.03E-27	5.24E-28	-0.170947282	AMG.706	AMG.706****	Sensitive to high-risk group
KU.55933	5.107	5.158	1.26E-26	1.18E-27	-0.072779917	KU.55933	KU.55933****	Sensitive to high-risk group
AICAR	8.133	7.979	9.61E-25	9.75E-26	0.214693732	AICAR	AICAR****	Sensitive to low-risk group
BIRB.0796	5.887	5.884	1.60E-24	1.74E-25	0.004306109	BIRB.0796	BIRB.0796****	Sensitive to low-risk group
NSC.87877	6.775	6.852	1.88E-24	2.17E-25	-0.09016	NSC.87877	NSC.87877****	Sensitive to high-risk

					9047			group
Vinblastine	-4.201	-4.22	3.95E-23	4.87E-24	0.024248777	Vinblastine	Vinblastine****	Sensitive to low-risk group
ABT.263	NA	3.174	7.15E-23	9.33E-24	NA	ABT.263	ABT.263****	NA
Tipifarnib	2.161	2.043	8.17E-23	1.12E-23	0.136834027	Tipifarnib	Tipifarnib****	Sensitive to low-risk group
Obatoclax.Mesylate	-1.015	-1.099	1.67E-22	2.42E-23	0.097934527	Obatoclax.Mesylate	Obatoclax.Mesylate****	Sensitive to low-risk group
CGP.60474	-1.668	-1.935	2.73E-22	4.16E-23	0.333924798	CGP.60474	CGP.60474****	Sensitive to low-risk group
BMS.708163	4.93	5.009	4.66E-22	7.42E-23	-0.110849471	BMS.708163	BMS.708163****	Sensitive to high-risk group
PF.562271	2.763	2.665	5.55E-22	9.65E-23	0.113876501	PF.562271	PF.562271****	Sensitive to low-risk group
EHT.1864	4.647	4.692	5.55E-22	9.45E-23	-0.067964856	EHT.1864	EHT.1864****	Sensitive to high-risk group
PAC.1	3.574	3.526	2.99E-21	5.41E-22	0.07366163	PAC.1	PAC.1****	Sensitive to low-risk group
OSI.906	3.657	3.803	3.80E-21	7.44E-22	-0.184210659	OSI.906	OSI.906****	Sensitive to high-risk group
Pyrimethamine	4.546	4.419	3.80E-21	7.21E-22	0.19327432	Pyrimethamine	Pyrimethamine****	Sensitive to low-risk group
IPA.3	5.777	5.767	2.24E-20	4.54E-21	0.009888303	IPA.3	IPA.3****	Sensitive to low-risk group
PHA.665752	4.932	5.067	3.07E-20	6.45E-21	-0.171347786	PHA.665752	PHA.665752****	Sensitive to high-risk group
GDC0941	2.137	2.269	3.77E-	8.20E-	-0.18552	GDC0941	GDC0941**	Sensitive to high-risk

			20	21	1661		**	group
GSK.650394	4.108	4.086	3.84E-20	8.62E-21	0.028787367	GSK.650394	GSK.650394****	Sensitive to low-risk group
MK.2206	2.783	2.658	1.02E-19	2.38E-20	0.150798359	MK.2206	MK.2206****	Sensitive to low-risk group
Lapatinib	4.448	4.313	1.72E-19	4.12E-20	0.153574958	Lapatinib	Lapatinib****	Sensitive to low-risk group
NU.7441	3.493	3.594	2.29E-19	5.64E-20	-0.171941553	NU.7441	NU.7441****	Sensitive to high-risk group
BIBW2992	2.287	2.202	5.72E-19	1.45E-19	0.088998904	BIBW2992	BIBW2992****	Sensitive to low-risk group
AS601245	3.179	3.175	1.37E-18	3.58E-19	0.004102709	AS601245	AS601245****	Sensitive to low-risk group
Salubrinal	4.138	4.01	1.92E-18	5.15E-19	0.174556551	Salubrinal	Salubrinal****	Sensitive to low-risk group
VX.680	2.655	2.737	5.25E-18	1.45E-18	-0.119400268	VX.680	VX.680****	Sensitive to high-risk group
AKT.inhibitor.VIII	3.53	3.528	5.67E-18	1.60E-18	0.002550796	AKT.inhibitor.VIII	AKT.inhibitor.VIII****	Sensitive to low-risk group
DMOG	6.608	6.491	7.46E-18	2.16E-18	0.113514501	DMOG	DMOG****	Sensitive to low-risk group
PD.0332991	2.324	2.261	1.76E-17	5.24E-18	0.083254371	PD.0332991	PD.0332991****	Sensitive to low-risk group
RO.3306	4.705	4.614	1.43E-16	4.36E-17	0.096534153	RO.3306	RO.3306****	Sensitive to low-risk group
WH.4.023	4.064	4.392	5.33E-16	1.66E-16	-0.490717208	WH.4.023	WH.4.023****	Sensitive to high-risk group
Epothilone	-4.935	-5.183	6.05E-	1.93E-	0.29299	Epothilone	Epothilone.B	Sensitive to

.B			16	16	2796	.B	****	low-risk group
SB590885	4.99	5.047	7.63E-16	2.49E-16	-0.073280619	SB590885	SB590885****	Sensitive to high-risk group
Sunitinib	3.325	3.192	1.71E-15	5.71E-16	0.1245959	Sunitinib	Sunitinib****	Sensitive to low-risk group
Pazopanib	4.495	4.572	5.13E-15	1.75E-15	-0.124510815	Pazopanib	Pazopanib****	Sensitive to high-risk group
Etoposide	1.623	1.501	5.24E-15	1.82E-15	0.149260423	Etoposide	Etoposide****	Sensitive to low-risk group
Doxorubicin	-1.872	-2.035	2.26E-14	8.18E-15	0.197981404	Doxorubicin	Doxorubicin****	Sensitive to low-risk group
Embelin	2.889	2.81	2.26E-14	8.05E-15	0.101093848	Embelin	Embelin****	Sensitive to low-risk group
Cisplatin	3.388	3.486	4.33E-14	1.60E-14	-0.123422106	Cisplatin	Cisplatin****	Sensitive to high-risk group
PD.173074	4.058	4.184	5.40E-14	2.03E-14	-0.193937973	PD.173074	PD.173074****	Sensitive to high-risk group
Rapamycin	-0.2327	-0.4038	6.09E-14	2.34E-14	0.246104971	Rapamycin	Rapamycin****	Sensitive to low-risk group
Shikonin	0.172	0.1666	7.15E-14	2.80E-14	0.006464249	Shikonin	Shikonin****	Sensitive to low-risk group
Gemcitabine	-2.381	-2.589	1.46E-13	5.81E-14	0.266908964	Gemcitabine	Gemcitabine****	Sensitive to low-risk group
MS.275	0.8052	0.7801	1.00E-12	4.07E-13	0.029399772	MS.275	MS.275****	Sensitive to low-risk group
WO2009093972	2.972	3.106	1.80E-12	7.45E-13	-0.21034729	WO2009093972	WO2009093972****	Sensitive to high-risk group

XMD8.85	3.28	3.18	2.70E-12	1.14E-12	0.116450489	XMD8.85	XMD8.85****	Sensitive to low-risk group
BX.795	2.136	2.292	7.18E-12	3.07E-12	-0.232022866	BX.795	BX.795****	Sensitive to high-risk group
AG.014699	4.177	4.265	1.87E-11	8.14E-12	-0.145941787	AG.014699	AG.014699****	Sensitive to high-risk group
AZD.0530	4.125	4.297	2.20E-11	9.72E-12	-0.263226274	AZD.0530	AZD.0530****	Sensitive to high-risk group
AUY922	-2.656	-2.766	2.33E-11	1.05E-11	0.130829061	AUY922	AUY922****	Sensitive to low-risk group
S.Trityl.L.cysteine	1.564	1.501	2.34E-11	1.07E-11	0.086495005	S.Trityl.L.cysteine	S.Trityl.L.cysteine****	Sensitive to low-risk group
WZ.1.84	5.667	5.617	4.98E-11	2.31E-11	0.065331502	WZ.1.84	WZ.1.84****	Sensitive to low-risk group
BMS.754807	1.216	1.33	6.18E-11	2.91E-11	-0.145622103	BMS.754807	BMS.754807****	Sensitive to high-risk group
Mitomycin.C	-1.008	-1.041	9.81E-11	4.69E-11	0.068448508	Mitomycin.C	Mitomycin.C****	Sensitive to low-risk group
Z.LLNle.CHO	2.658	2.481	1.26E-10	6.10E-11	0.245260797	Z.LLNle.CHO	Z.LLNle.CHO****	Sensitive to low-risk group
FH535	2.146	2.028	1.61E-10	7.92E-11	0.168636011	FH535	FH535****	Sensitive to low-risk group
JNJ.26854165	2.898	2.963	2.56E-10	1.28E-10	-0.102928087	JNJ.26854165	JNJ.26854165****	Sensitive to high-risk group
PF.4708671	4.955	4.95	4.57E-09	2.32E-09	0.007772804	PF.4708671	PF.4708671****	Sensitive to low-risk group

Bicalutamide	4.516	4.547	5.87E-09	3.02E-09	-0.050997118	Bicalutamide	Bicalutamide****	Sensitive to high-risk group
FTI.277	3.741	3.674	1.31E-08	6.82E-09	0.07042685	FTI.277	FTI.277****	Sensitive to low-risk group
CCT018159	3.163	3.201	1.61E-08	8.50E-09	-0.069296468	CCT018159	CCT018159****	Sensitive to high-risk group
VX.702	4.349	4.408	1.75E-08	9.39E-09	-0.109530068	VX.702	VX.702****	Sensitive to high-risk group
Cyclopamine	6.491	6.456	2.52E-08	1.37E-08	0.023739945	Cyclopamine	Cyclopamine****	Sensitive to low-risk group
CGP.082996	4.062	3.975	3.60E-08	1.98E-08	0.075794055	CGP.082996	CGP.082996****	Sensitive to low-risk group
BI.D1870	2.553	2.626	6.67E-08	3.72E-08	-0.099176258	BI.D1870	BI.D1870****	Sensitive to high-risk group
AZD.2281	4.821	4.899	7.60E-08	4.29E-08	-0.114635001	AZD.2281	AZD.2281****	Sensitive to high-risk group
RDEA119	2.392	2.525	1.32E-07	7.58E-08	-0.222942624	RDEA119	RDEA119****	Sensitive to high-risk group
ABT.888	5.341	5.303	1.44E-07	8.32E-08	0.053246814	ABT.888	ABT.888****	Sensitive to low-risk group
JW.7.52.1	-1.658	-1.796	2.15E-07	1.26E-07	0.214813283	JW.7.52.1	JW.7.52.1****	Sensitive to low-risk group
Thapsigargin	-4.17	-4.31	6.21E-07	3.69E-07	0.129471199	Thapsigargin	Thapsigargin****	Sensitive to low-risk group
A.770041	3.922	4.007	1.16E-06	6.99E-07	-0.186055354	A.770041	A.770041****	Sensitive to high-risk group

NVP.BEZ235	-2.406	-2.383	1.34E-06	8.14E-07	-0.044775363	NVP.BEZ235	NVP.BEZ235****	Sensitive to high-risk group
Nilotinib	4.247	4.287	1.47E-06	9.06E-07	-0.073674169	Nilotinib	Nilotinib****	Sensitive to high-risk group
AP.24534	1.626	1.628	2.43E-06	1.51E-06	-0.003776129	AP.24534	AP.24534****	Sensitive to high-risk group
Bleomycin	1.619	1.524	3.06E-06	1.93E-06	0.139445898	Bleomycin	Bleomycin****	Sensitive to low-risk group
Nutlin.3a	4.751	4.774	4.24E-06	2.70E-06	-0.057921049	Nutlin.3a	Nutlin.3a****	Sensitive to high-risk group
Bortezomib	-5.195	-5.333	5.15E-06	3.32E-06	0.268662872	Bortezomib	Bortezomib****	Sensitive to low-risk group
Dasatinib	2.249	2.483	5.58E-06	3.64E-06	-0.393917359	Dasatinib	Dasatinib****	Sensitive to high-risk group
QS11	3.78	3.777	9.52E-06	6.28E-06	0.003303753	QS11	QS11****	Sensitive to low-risk group
SB.216763	5.552	5.554	1.50E-05	1.00E-05	-0.001836029	SB.216763	SB.216763****	Sensitive to high-risk group
NVP.TAE684	1.711	1.795	1.98E-05	1.34E-05	-0.14391785	NVP.TAE684	NVP.TAE684****	Sensitive to high-risk group
CMK	3.687	3.634	2.07E-05	1.41E-05	0.048275996	CMK	CMK****	Sensitive to low-risk group
Gefitinib	2.03	1.986	2.55E-05	1.76E-05	0.033770744	Gefitinib	Gefitinib****	Sensitive to low-risk group
CI.1040	2.617	2.671	2.83E-05	1.97E-05	-0.08730	CI.1040	CI.1040****	Sensitive to high-risk

					3915			group
GW843682X	-2.324	-2.358	3.24E-05	2.28E-05	0.044624411	GW843682X	GW843682X****	Sensitive to low-risk group
CEP.701	-0.2609	-0.2079	4.85E-05	3.48E-05	-0.068541086	CEP.701	CEP.701****	Sensitive to high-risk group
JNK.Inhibitor.VIII	5.871	5.882	4.85E-05	3.48E-05	-0.007959303	JNK.Inhibitor.VIII	JNK.Inhibitor.VIII****	Sensitive to high-risk group
PF.02341066	4.371	4.388	7.17E-05	5.20E-05	-0.0212342	PF.02341066	PF.02341066****	Sensitive to high-risk group
Erlotinib	4.608	4.572	0.000118814	8.71E-05	0.047945465	Erlotinib	Erlotinib****	Sensitive to low-risk group
KIN001.135	6.117	6.104	0.000118814	8.78E-05	0.016183006	KIN001.135	KIN001.135****	Sensitive to low-risk group
Camptothecin	-4.081	-4.043	0.000143803	0.000107331	-0.049559594	Camptothecin	Camptothecin***	Sensitive to high-risk group
Temsirolimus	-1.129	-1.11	0.000177434	0.000133719	-0.050590551	Temsirolimus	Temsirolimus***	Sensitive to high-risk group
Paclitaxel	-2.871	-2.951	0.000196377	0.000149417	0.128176607	Paclitaxel	Paclitaxel***	Sensitive to low-risk group
TW.37	-0.6771	-0.6231	0.00020211	0.000155244	-0.149009832	TW.37	TW.37***	Sensitive to high-risk group
Axitinib	3.366	3.384	0.000317376	0.000246081	-0.030514043	Axitinib	Axitinib***	Sensitive to high-risk group
GDC.0449	5.726	5.731	0.00089476	0.000700247	-0.00499091	GDC.0449	GDC.0449***	Sensitive to high-risk group

SL.0101.1	5.612	5.634	0.001493921	0.001179981	-0.036072029	SL.0101.1	SL.0101.1**	Sensitive to high-risk group
Vorinostat	1.203	1.232	0.001715661	0.001367556	-0.037412076	Vorinostat	Vorinostat**	Sensitive to high-risk group
Sorafenib	3.873	3.873	0.006029933	0.004850164	0.00033499	Sorafenib	Sorafenib**	Sensitive to low-risk group
Docetaxel	-5.343	-5.362	0.007150148	0.005803019	0.030072178	Docetaxel	Docetaxel**	Sensitive to low-risk group
Elesclomol	-2.965	-2.91	0.008058582	0.006598694	-0.074198385	Elesclomol	Elesclomol**	Sensitive to high-risk group
ATRA	5.209	5.242	0.018485882	0.015270946	-0.073462338	ATRA	ATRA*	Sensitive to high-risk group
BI.2536	-1.599	-1.625	0.027048302	0.022540251	0.052602631	BI.2536	BI.2536*	Sensitive to low-risk group
BMS.536924	2.629	2.596	0.030182789	0.02537104	0.03017486	BMS.536924	BMS.536924*	Sensitive to low-risk group
