# Supplementary material for: Prognostic integration of tumor microenvironment and parthanatos-related genes in gastric cancer: a machine learning-driven risk model and immune landscape profiling
Source: Front Immunol. 2026 Feb 20;17:1636331. doi: 10.3389/fimmu.2026.1636331 (PMC12962909; doi:10.3389/fimmu.2026.1636331)
Supplement: Supplementary file 2 [file DataSheet2.pdf]

# Appendix: Survival Feature Selection and Modeling Methods Explanation

This paper adopts a two-stage process for survival feature selection and modeling: the first stage is feature selection (Variable mode), and the second stage uses only the selected features to fit the model. The theoretical benefits of this process are mainly reflected in three aspects:

**1. Bias-Variance Tradeoff**

In the selection stage, dimensionality is reduced, and variance is reduced through strong regularization (such as Lasso/Elastic Net) or structural ranking (such as RSF minimal depth, GBM importance).

In the modeling stage, more flexible families of functions (such as CoxBoost, GBM, RSF) are used to capture non-linearity or complex interactions, thereby reducing bias.

**2. Robustness and Interpretability**

Statistical interpretability rules are first used to select features (such as Stepwise Cox's AIC, Elastic Net's non-zero coefficients, SuperPC's thresholds), and then complex models are used for fitting, preventing noisy variables from entering non-linear models and improving robustness.

**3. Phenotypic Heterogeneity and Correlated Structure**

For example, RSF's splitting criterion is used for rough screening sensitive to non-linearity/interactions, and then the linear Cox model is used for robust estimation, separating the "non-linearity detection" and "effect estimation" processes.

Below is an explanation of each feature selection method, including principles, implementation steps, key parameter settings, and tuning strategies.

**1. Elastic Net / Lasso / Ridge (Cox-based Penalized Regression)**

**Principle:** In the Cox proportional hazards framework, variables are selected and shrunk using a penalty term:

Lasso (L1) compresses irrelevant coefficients to 0;

Ridge (L2) does continuous shrinkage;

Elastic Net is a convex combination of L1/L2, balancing sparsity and group effects.

**Implementation Steps:**

Use expression matrix X and survival outcomes (time, status) to fit glmnet(..., family="cox").

Perform 10-fold cross-validation to get lambda.min.

Select non-zero coefficients as final features.

**Key Parameters:**

alpha: Lasso=1, Ridge=0, Elastic Net can be chosen from preset candidate values (e.g., Enet[alpha=0.4]).

lambda: Automatically selected by CV as lambda.min.

nfolds=10, glmnet standardizes each column by default.

**Tuning Strategy:** The alpha for Elastic Net is determined by enumerating candidate values, and lambda is automatically selected by 10-fold CV, with no additional grid

search.

**2. Stepwise Cox (Stepwise Regression)**
**Principle:** Forward/backward/bidirectional stepwise variable addition or removal based on AIC to get the AIC-optimal model.
**Implementation Steps:**
Fit initial Cox or null model.
Call step() to add/remove variables according to AIC until AIC no longer improves.
**Key Parameters:**
direction: forward/backward, specified by combination string.
Other settings are default for R (trace=0 for silent running).
**Tuning Strategy:** AIC automatically decides the subset, and no outer search is performed.

**3. CoxBoost**
**Principle:** A stepwise gradient boosting method within the Cox framework, updating coefficients step by step and applying penalties to control complexity and avoid overfitting.
**Implementation Steps:**
Estimate penalties using optimCoxBoostPenalty(start.penalty=500).
Use cv.CoxBoost(K=10, type="verweij", maxstepno=500) to select the optimal step number.
Retrain CoxBoost with the optimal number of steps, and non-zero coefficients are used as features.
**Key Parameters:**
Penalty term estimated adaptively from the data.
CV: 10-fold, type="verweij", maximum steps = 500.

**4. SuperPC (Supervised Principal Component Analysis for Survival)**
**Principle:** A statistical score is calculated for each gene based on its association with the outcome, and high-scoring genes are selected to form supervised principal components for survival prediction.
**Implementation Steps:**
Train feature scores using superpc.train(type="survival", s0.perc=0.5).
Select the best threshold using superpc.cv(n.threshold=20, n.fold=10, n.components=3).
Select features with |feature score| > 0.5 for modeling.
**Key Parameters:**
s0.perc=0.5
n.threshold=20
n.fold=10
n.components=3

**5. plsRcox (Partial Least Squares + Cox)**

**Principle:** PLS extracts latent components related to survival outcomes and is used in the Cox model; the sparse version can be used for variable selection.
**Implementation Steps:**
Use cv.plsRcox to choose the number of components, nt.
Fit the final model with sparse=TRUE, and non-zero coefficients are used as features.
**Key Parameters:**
Number of components nt automatically selected by CV.
sparse=TRUE.

**6. RSF (Random Survival Forest)**
**Principle:** A random forest for survival outcomes, using the log-rank splitting criterion to minimize heterogeneity between groups. Feature importance or minimal depth can be used for selection.
**Implementation Steps:**
Build RSF: rfsrc(Surv(time, status)~., ntree=1000, nodesize=5, splitrule="logrank", importance=TRUE)
Use var.select to select topvars as features.
**Key Parameters:**
ntree=1000, nodesize=5, splitrule="logrank", importance=TRUE

**7. survivalSVM (Survival Support Vector Machine)**
**Principle:** SVM ranking approach for survival data modeling, generating risk scores; no sparsification, retains the original feature set.
**Implementation Steps:**
survivalsvm(Surv~., gamma.mu=1, opt.meth="ipop")
The feature selection phase directly returns the input variables.
**Key Parameters:**
gamma.mu=1, opt.meth="ipop"

**8. GBM (Cox-distributed Gradient Boosting Trees)**
**Principle:** Using Cox deviation as the loss function, small trees are added to fit residuals, and feature importance is calculated for selection.
**Implementation Steps:**
Train CV: n.trees=100000, interaction.depth=3, n.minobsinnode=10, shrinkage=0.001, cv.folds=10
Retrain final model based on the optimal number of trees.
Select features with relative importance >0.
**Key Parameters:**
Distribution: coxph
n.trees=100000 (during CV)
interaction.depth=3
n.minobsinnode=10
shrinkage=0.001
cv.folds=10

**Unified Explanation and Two-Stage Process**

1. Two-Stage Approach: First select features, then model, ensuring only robust features are used in the modeling stage.
2. Feature Output: Each method returns non-zero or important features according to the rules above; if fewer than 2 features are retained, the combination is excluded from modeling.
3. Modeling and Evaluation: Fit the model using the selected features, compute risk scores, and then calculate Harrell's C-index on the queue using Surv(time, status)~RS.
4. Parameter Adjustment Summary: Except for Elastic Net's alpha (determined by candidate enumeration) and key hyperparameters selected by internal CV/AIC in each method, no additional grid or random search is performed to ensure reproducibility and manageable computational costs.

**Example Combinations and Applications:**

**Enet(Selection) + CoxBoost(Model):** Enet removes redundant collinear variables, while CoxBoost captures weak non-linearity and non-additivity.

**RSF(Selection) + Enet(Model):** RSF is sensitive to non-linearity/interactions, while Enet performs robust linear estimation.

**GBM(Selection) + Ridge(Model):** GBM removes weak signals, while Ridge retains group-related structures to suppress variance.

**StepCox(Selection) + RSF(Model):** AIC retains statistically interpretable variables, while RSF absorbs potential non-linearity.
